# Nanoparticle-Induced Cross-Tolerance: A Review of Mechanisms for Concurrent Biotic and Abiotic Stress Mitigation in Crops

**DOI:** 10.3390/plants15091334

**Published:** 2026-04-27

**Authors:** Mukhtar Iderawumi Abdulraheem, Iram Naz, Marissa Pérez-Alvarez, Jiandong Hu, Gregorio Cadenas-Pliego, Olaniyi Amos Fawole

**Affiliations:** 1 Postharvest and Agroprocessing Research Centre, Department of Botany and Plant Biotechnology, University of Johannesburg, Auckland Park, Johannesburg 2006, South Africa; 2 South African Research Chairs Initiative in Sustainable Preservation and Agroprocessing Research, Faculty of Science, University of Johannesburg, Auckland Park, Johannesburg 2006, South Africa; 3 Department of Electrical Engineering, Henan Agricultural University, Zhenghou 450002, China; jdhu@henau.edu.cn; 4 Department of Botany, The Government Sadiq College Women University, Bahawalpur 63100, Pakistan; iramnaz4545@gmail.com; 5 Centro de Investigación en Química Aplicada, Blvd. Enrique Reyna 140, Saltillo 25294, Coahuila, Mexico; marissa.perez@ciqa.edu.mx

**Keywords:** nanoparticles, cross-tolerance, biotic and abiotic stress, antioxidant defense, phytohormone signaling, crop resilience, sustainable agriculture

## Abstract

Plants in agricultural systems rarely face single stressors; instead, they encounter concurrent biotic (pathogen, pests) and abiotic (drought, salinity, heavy metals) stresses that causes severely reduce crop yields and endanger food security. The traditional methods of breeding, genetic engineering, and agrochemicals tend to target individual stresses and still do not suffice in the complex field conditions. Compared to these approaches, nanotechnology offers distinct advantages: nanoparticles (NPs) can be applied as foliar sprays or seed treatments without lengthy breeding cycles or regulatory hurdles associated with genetically modified organisms. However, nanotechnology is not inherently “better” but rather complementary to crop engineering; each approach has specific strengths. Breeding and genetic engineering provide heritable, long-term solutions, while nanotechnology offers immediate, season-specific, and reversible interventions. Cross-tolerance, the phenomenon whereby exposure to one stress enhances tolerance to another, offers a promising alternative. This review critically examines how NPs act as stress-priming agents that induce cross-tolerance by activating overlapping defense networks, including antioxidant systems (SOD, CAT, APX), phytohormonal crosstalk (ABA, SA, JA), osmolyte homeostasis, and stress-responsive gene expression. We synthesize current evidence on NP uptake, translocation, and cellular interactions, and evaluate their dual role in directly suppressing pathogens while simultaneously enhancing plant immune responses and physiological resilience. However, efficacy is highly dose-dependent: low, subtoxic doses prime defense through hermetic ROS signaling, whereas supraoptimal doses cause phytotoxicity. The current challenges in nano-mediated stress alleviation include: (i) a persistent laboratory-to-field translation gap, with field outcomes averaging only 60–70% of greenhouse efficacy; (ii) dose-dependent phytotoxicity; (iii) poor reproducibility across studies; (iv) scalability and formulation stability issues; and (v) insufficient understanding of long-term environmental fate, including soil accumulation, non-target organism effects, and food chain safety. Future research should consider field-validated formulations (e.g., SiNPs, ZnONPs, Fe_3_O_4_NPs) across major staple crops); integrating nanotechnology with precision agriculture through nanosensors, remote sensing, and artificial intelligence for site-specific, dose-optimized applications;developing smart, biodegradable nanoparticles with stimuli-responsive release; and establishing harmonized regulatory frameworks for nano-agrochemical approval. When deployed responsibly, nanoparticle-induced cross-tolerance represents a sustainable approach to improve crop resistance against multifactorial stress, with significant implications for climate-resilient agriculture and global food security.

## 1. Introduction

The combination of biotic and abiotic stresses is threatening global agriculture and significantly decreasing crop productivity and endangering global food security [1]. Globally, agricultural systems face a wide range of environmental limitations that can be broadly divided into biotic stresses, which include pathogenic organisms like bacteria, fungi, viruses, and pests like insects and nematodes, and abiotic stresses such as drought, salinity, extreme temperatures, heavy metal pollution, flooding, and nutrient depletion [2]. Each of these stressors may have pronounced negative effects on plant development and production, but when they combine, they cause complex stress conditions that are much more devastating than those caused by individual stresses. Biotic stresses contribute to significant losses in crops annually, even after the massive application of pesticides and other control measures [3]. The invasion of the pathogens leads to the immune responses of the plants, and in most cases, the natural defense mechanisms are stressed to the extent of failure. Meanwhile, the abiotic stresses like drought and salinity interfere with water relations, photosynthesis, ion homeostasis, and metabolic balance [4]. The problem of climate change exacerbates these conditions by exposing people to more heat waves, unpredictable rainfalls, salinization of soils, and heavy metals in the soil as a result of human activities and inappropriate farming methods. The interaction of various types of stresses, including drought and pathogen infections or salinity and pest attacks are a new and complex challenge to plant stress and agro sustainability [5].

Nanoparticles (NPs) are defined as materials with at least one dimension between 1 and 100 nanometers [6]. At this scale, materials exhibit unique physicochemical properties that differ significantly from their bulk counterparts. The extremely high surface-to-volume ratio of NPs enhances their reactivity and interaction with biological membranes [7,8]. This property has been confirmed by multiple independent studies using surface resonance and atomic force microscopy. Surface charge (zeta potential) influences NP stability, aggregation, and binding to plant cell walls, with negatively charged NPs often showing different uptake patterns than positively charged ones [9]. Quantum confinement effects alter the optical and electronic properties of NPs, enabling applications such as nanosensors and photocatalytic activity [10]. Shape and morphology (spheres, rods, tubes, sheets) affect cellular uptake, transport, and biological activity; for example, carbon nanotubes exhibit different behavior compared to spherical metal NPs [11]. Many metal and metal oxide NPs (e.g., ZnONPs, CuONPs) slowly release ions in aqueous environments, a property critical for their function as nanofertilizers and antimicrobial agents [12]. NPs tend to aggregate in solution due to van der Waals forces, and surface coatings are often required to maintain stability [13]. These properties directly determine how NPs enter plants, translocate to target tissues, and exert their stress-protective effects.

Traditional methods of stress management, such as breeding, genetic engineering, and agrochemicals, have made a significant contribution to crop tolerance but faces limitations under simultaneous multiple stressors [14]. Breeding programs have lengthy breeding cycles and can focus on particular characteristics rather than on a general tolerance [7]. Genetic engineering practices can be confronted with regulatory challenges and acceptance problems. The use of agrochemicals is dangerous to the environment, and it contributes to the poor health of the soil, possibly causing pathogen and pest resistance [15]. Thus, novel, sustainable, and integrative solutions that promote the resilience of plants in the multifactorial stressful situations are urgently required [10]. Nanotechnology is a new disruptive technology that has great potential to transform agriculture in recent decades. Nanoparticles (NPs), which are substances with a minimum of one dimension in the nanoscale (1–100 nm), are characterized by special physicochemical properties, such as a high surface-to-volume ratio, increased reactivity, controllable surface charge, and quantum confinement [16,17]. These properties allow the nanoparticles to act upon plant systems in a set of new ways, shaping the physiological, biochemical, and molecular processes more effectively than their bulk counterparts. Agricultural applications of nanotechnology include nano-fertilizers, nano-pesticides, nano-herbicides, nanosensors, and nano-enabled delivery systems. Recent comprehensive reviews have reinforced the promise of nanotechnology for enhancing abiotic stress tolerance in plants. Azameti and Imoro [18] provided a critical assessment of how nanoparticles improve plant resilience through modulation of antioxidant systems, osmotic adjustment, and nutrient uptake, while highlighting the need for standardized application protocols. Their analysis supports the central premise of the current review that nanotechnology offers a viable pathway for mitigating climate-induced agricultural stresses. In addition to their nutrient carrier or antimicrobial properties, nanoparticles have proven to be signaling modulators and priming agents that promote innate plant defense [9]. Their size allows them to enter through plant surfaces (stomata, cuticular pores, and epidermal cells of roots), and subsequently translocate to other plant organs via the apoplastic and symplastic routes [19].

Among the uses of nano-agriculture is the induction of cross-tolerance in crops, which is one of the most promising. Cross-tolerance describes a physiological process in which exposure to a given form of stress increases the tolerance of the plant to another form of stress, which, in most cases, is not related to the first [16]. This adaptive mechanism is mediated to a great extent by common defense mechanisms and overlapping signaling pathways. There is emerging evidence indicating that exposure to nanoparticles can induce regulated ROS generation, causing activation of antioxidant defense systems; superoxide dismutase (SOD), catalase (CAT), peroxidases (POD), and ascorbate -glutathione cycle [20]. At the same time, nanoparticles regulate the phytohormonal balance, increase the accumulation of osmolytes (e.g., proline, glycine betaine), boost the synthesis of secondary metabolites (phenolics, flavonoids, lignin), and adjust the expression of stress-sensitive genes [21]. These combined responses enable nanoparticles to boost tolerance to adverse abiotic pressure, including drought, salinity, heavy metal toxicity, and heat stress, as well as increasing resistance towards pathogens and pests [10]. As an example, nanoparticles of metal and metal oxides have been demonstrated to strengthen cell wall engineering, lignify, and provide systemic acquired resistance (SAR), which restricts pathogen growth. Simultaneously, they enhance photosynthetic efficiency, stabilize membranes, control ion homeostasis, and increase water-use efficiency in abiotic stress situations [22].

While individual studies have demonstrated NP-mediated stress mitigation, no comprehensive synthesis has specifically focused on the mechanisms of cross-tolerance -how NPs enable simultaneous resistance to biotic and abiotic stress. This review fills the gap by providing a mechanistic framework linking NP uptake, redox regulation, hormonal crosstalk, and gene expression dynamics to the induction of cross-tolerance. We discuss the NP uptake, translocation, cellular interactions, redox regulation, hormonal crosstalk, and gene expression dynamics, synthesizing recent experimental results and conceptual frameworks that explain how nano-enabled solutions can be used to increase crop resilience under increasing environmental pressures. Despite the promising potential, several limitations temper enthusiasm. NP behavior varies dramatically with size, shape, surface charge, and concentration—effects observed at 50 mg/L may reverse at 200 mg/L. Dose dependency is critical: low, subtoxic doses prime defenses through hormetic ROS signaling, whereas supraoptimal doses cause phytotoxicity, growth inhibition, and cellular damage. Environmental risks—soil accumulation, effects on non-target organisms, and potential food chain transfer—remain poorly characterized. Moreover, most studies are conducted under controlled greenhouse conditions; field validation is scarce, with fewer than 15% of published studies including field trials.

Concrete examples in specific crop–stress combinations remain limited. For instance, silicon nanoparticles have been shown to concurrently enhance drought tolerance and resistance against blast disease in rice (*Oryza sativa*), while zinc oxide nanoparticles mitigate cadmium toxicity and bacterial wilt in tomato (*Solanum lycopersicum*). However, a major bottleneck for field application is the lack of efficient and targeted delivery systems that ensure nanoparticle bioavailability while minimizing off-target effects. In this context, emerging nano-delivery platforms—such as nanoliposomes—offer significant advantages by encapsulating bioactive compounds, protecting them from premature degradation, facilitating cellular uptake, and enabling controlled release. Also, concrete criticisms remain that most studies lack validation in real-field agronomic settings, and the long-term fate, biodegradability, and potential ecotoxicity of nanocarriers in soil-plant systems are poorly understood [23]. Addressing these gaps will be essential to translate nanoparticle-induced cross-tolerance from proof-of-concept to sustainable agricultural practice. This review critically examines both the promise and the limitations of nanoparticle-induced cross-tolerance, emphasizing where evidence is robust and where gaps remain, with the goal of informing future research toward climate-resilient agriculture and global food security.

This review advances understanding of nanotechnology applications in plant stress tolerance through five specific contributions: (i) it provides the first comprehensive mechanistic framework specifically focused on cross-tolerance—the ability of nanoparticles to confer simultaneous resistance to both biotic and abiotic stresses—rather than treating these stress categories separately, (ii) it quantitatively analyzes the laboratory-to-field translation gap, documenting that field efficacy averages only 60–70% of greenhouse results across multiple nanoparticle types and crop species, (iii) it introduces a multiscale framework connecting molecular mechanisms (ROS signaling, hormonal crosstalk, gene regulation) to cellular, whole-plant, field, and ecosystem outcomes, enabling integrated assessment of nanoparticle effects, (iv) it provides economic feasibility analysis with return on investment calculations for major nanoparticle types, identifying SiNPs and ZnONPs as commercially viable with estimated return on investment (ROI) values of 83–280× (where ROI = [net benefit ÷ cost] × 100%, expressed as a multiplier of the initial investment; e.g., 100× means that $1 invested returns $100 in net benefit), based on field-validated yield increases in wheat and rice while demonstrating that AgNPs and CNTs remain economically unviable, and (v) it critically assesses translational hurdles across production, formulation, regulatory, economic, and social dimensions, with specific progress markers and actionable recommendations. Together, these contributions distinguish this review from existing literature, which has largely focused on individual stress responses, qualitative descriptions, or laboratory-scale studies without critical integration of economic and translational perspectives.

## 2. Nanoparticle Uptake, Translocation, and Interaction in Plants

### 2.1. Entry Pathways

**Figure 1:**
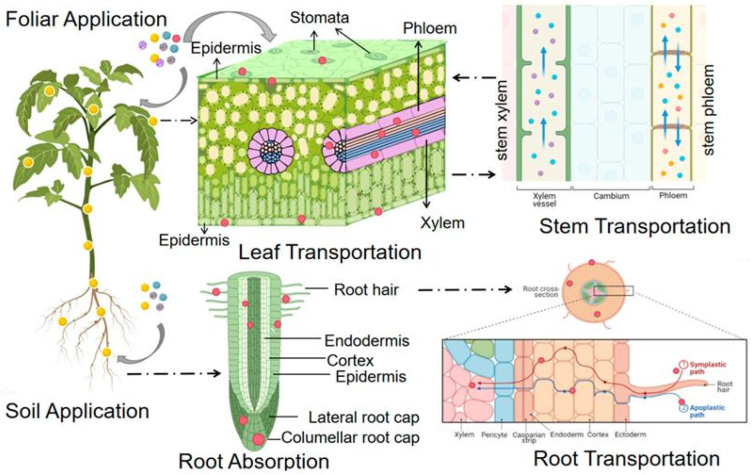
A schematic diagram of the uptake and translocation of NPs in plants through foliar application or root exposure treatment [26].

Nanoparticles into plants can occur via different routes, such as the roots, leaves, and even seeds, and the exact route of entry of the NP may, in many cases, depend on physicochemical characteristics of the NP and the physiological condition of a plant [24]. For small nanoparticles (<20 nm), transport mechanisms largely resemble those of dissolved mineral ions and compounds, including passive diffusion, facilitated transport via aquaporins or ion channels, and endocytosis [25]. The entry route determines further distribution, accumulation, and ultimate biological effects [19]. Understanding these pathways is essential for predicting NP behavior and optimizing agricultural application. The pathways of nanoparticles’ entry are illustrated in Figure 1.

#### 2.1.1. Root Uptake Mechanisms

Roots are the primary entry route for NPs applied to soil or hydroponic systems. NPs cross the root epidermis and cortex through several mechanisms: passive diffusion through cell wall pores (for smaller NPs, typically <10 nm); facilitated transport via aquaporins or ion channels; and endocytosis, which enables internalization of larger particles [25,27]. Surface charge influences uptake: negatively charged NPs interact differently with cell wall components than positively charged or neutral particles [28]. After entering root cells, NPs move radially toward the vascular cylinder, where they load into xylem for upward transport [20].

Apoplastic vs. symplastic transport: A key distinction is whether NPs move through apoplastic pathways (cell walls and intercellular spaces) or symplastic pathways (through plasmodesmata connecting living cells). The Casparian strip in the endodermis blocks apoplastic movement, forcing NPs into symplastic routes to access the xylem. This barrier significantly affects root-to-shoot translocation efficiency [29].

Active vs. passive uptake: While small NPs (<10 nm) may enter through passive diffusion, larger NPs typically require energy-dependent processes. Endocytosis—including clathrin-mediated, caveolae-dependent, and macropinocytosis pathways—is an active process that consumes cellular energy. However, the relative contribution of active vs. passive uptake for different NP types remains poorly quantified, with most evidence derived from pharmacological inhibition studies rather than direct observation [25].

#### 2.1.2. Foliar Uptake Mechanism

When used as foliar sprays, NPs are capable of getting deposited that way into plant leaves via stomata, hydathodes, and trichomes [4]. Stomatal entry is effective for NPs <15–40 nm, while the cuticle typically restricts uptake of particles >5 nm unless aided by surfactants or adjuvants [30]. Leaf surface wounds or micro-cracks provide additional entry points [31]. The cuticular pathway has both lipophilic and hydrophilic pathways, with NP surface chemistry determining which pathways dominates [24]. After crossing the cuticle and epidermis, NPs reach the palisade and spongy parenchyma before loading into phloem for systemic [32]. 

### 2.2. Translocation Pathways

After the absorption through the root or foliar route, nanoparticles are transported into the plant, a phenomenon called translocation, and their effectiveness in the agronomic setting depends on it [33]. Translocation efficiency and pattern depend on NP properties (size, shape, surface charge, composition) and plant characteristics (species, developmental stage, physiological status).

#### 2.2.1. Xylem Transport 

The nanoparticles that are absorbed by the roots are mainly carried upwards through the xylem and the stream of transpiration, to the stems and leaves [26]. The size exclusion limit of xylem—approximately 20–50 nm for most species—restricts larger NPs from efficient long-distance transport. However, the precise relationship between NP size and xylem loading efficiency remains incompletely characterized, with conflicting results across studies [34]. It is hypothesized that NP surface chemistry and protein corona formation may modulate xylem loading, but direct experimental evidence is lacking. 

#### 2.2.2. Phloem Transport

While in the vascular tissue, NPs may be load for long distances transport to sink organs (roots, fruits, developing leaves). Phloem mobility is particularly important for delivery of NPs to harvestable tissues (grains, fruits) and for foliar-applied NPs to reach roots [1,35]. The phloem mobility varies considerably among NP types: AgNPs and chitosan NPs are highly phloem-mobile due to their small size and surface charge, enabling accumulation in sink organs such as fruits and grains; in contrast, TiO_2_NPs and most metal oxide NPs (>30 nm) show limited phloem loading and remain predominantly in source leaves [29]. Notably, the molecular mechanisms of phloem loading and unloading for NPs remain speculative, with limited direct evidence for specific transporters or channels [8]. Carbon nanotubes exhibit intermediate phloem mobility, with transport efficiency depending on surface functionalization. These differences have direct agronomic consequences—for example, ZnONPs with high phloem mobility are preferred for grain biofortification, whereas CuONPs with limited phloem movement are better suited for foliar disease control without contaminating edible portions. 

#### 2.2.3. Cell-to-Cell Movement 

Cell-to-cell movement occurs through plasmodesmata—specialized channels connecting adjacent plant cells. These channels have size exclusion limits of approximately 3–50 nm, allowing smaller NPs to move symplastically [26]. However, the extent to which NPs utilize plasmodesmatal transport versus endocytosis-mediated vesicular transport remains controversial, with limited direct visualization studies [25]. Carbon nanotubes and small metal NPs (<20 nm) show evidence of plasmodesmatal movement, but larger NPs likely require endocytosis and vesicular trafficking [36].

### 2.3. Critical Assessment of Experimental Limitations

Several limitations constrain current understanding of NP uptake and translocation:

- Most studies use model NPs (e.g., spherical AgNPs, ZnONPs) under idealized conditions (deionized water, sterile media) that do not reflect the complexity of soil or leaf surfaces. NP behavior in real agricultural environments—where organic matter, salts, and microbial communities alter aggregation and surface chemistry—remains poorly characterized.
- Mechanisms such as aquaporin-mediated transport and plasmodesmatal movement are presented in the literature with confidence that exceeds experimental support. Direct evidence for NP passage through aquaporins is limited to a few studies using pharmacological inhibitors; molecular confirmation (e.g., transport assays with aquaporin-knockout mutants) is lacking. Similarly, plasmodesmatal movement has been inferred from co-localization studies rather than direct observation of NP passage.
- Active vs. passive uptake is often inferred from energy-depletion experiments (e.g., cold temperature, metabolic inhibitors) that can have off-target effects, confounding interpretation.
- Quantitative comparisons across studies are difficult due to inconsistent reporting of NP properties (size distribution in application medium, surface charge, aggregation state) and experimental conditions (pH, ionic strength, exposure duration).

To facilitate a comparative understanding of how different nanoparticle types behave in terms of uptake, translocation, and agricultural utility, Table 1 summarizes key characteristics of major NP classes relevant to crop stress mitigation. Aside qualitative descriptions, quantitative comparisons reveal significant differences with agronomic implications. The translocation factor (TF = concentration in shoot/concentration in root) varies widely: SiNPs and CNTs exhibit high TFs (>1.5), whereas CuONPs and TiO_2_NPs show low TFs (<0.5). Similarly, the percentage of applied dose reaching target tissues differs markedly—up to 60–80% of foliar-applied AgNPs may remain on leaf surfaces, while only 10–20% translocates systemically. Such differences are agronomically relevant: for biofortification, high phloem mobility and sink-organ accumulation are desirable; for surface-acting antimicrobial protection, limited systemic movement may be preferable to reduce off-target accumulation. Moreover, uptake efficiency is influenced by agronomic practices: seed priming achieves higher internalization of small NPs (<20 nm) into embryonic tissues, whereas foliar sprays are more effective for cuticle-penetrating NPs (CNTs, chitosan). For larger nanoparticles (50–100 nm), transport mechanisms differ significantly. These particles are generally excluded from symplastic movement due to plasmodesmata size exclusion limits (approximately 3–50 nm). Instead, they rely on apoplastic transport through cell wall pores and intercellular spaces, which is slower and less efficient. Endocytosis becomes the primary route for cellular internalization of large NPs, requiring energy and involving clathrin-mediated or caveolae-dependent pathways. Translocation to shoots is severely restricted for large NPs, with most remaining in root tissues or leaf application sites. For example, TiO_2_NPs (50–100 nm) show minimal xylem loading and accumulate primarily on leaf surfaces or in root apoplast [26,29].

### 2.4. Hierarchy of Evidence: Distinguishing Established, Postulated, and Hypothetical Mechanisms

Throughout this review, we employ a standardized terminology to distinguish the strength of evidence supporting each mechanistic claim. [Established] indicates mechanisms supported by direct experimental evidence from multiple independent studies, ideally including field validation, molecular confirmation (e.g., mutant studies, gene expression), or direct visualization (e.g., microscopy). [Postulated/Proposed] indicates mechanisms that are consistent with indirect evidence (e.g., pharmacological inhibition studies, correlative data) and mechanistically plausible but lacking direct molecular confirmation. [Hypothetical/Speculative] indicates mechanisms that are logically plausible based on first principles or extrapolation from other systems but for which no direct evidence exists in the context of nanoparticle-plant interactions. This classification is applied at the first mention of each major mechanism and summarized in Section 8.

## 3. Mechanisms of Nanoparticle-Induced Abiotic Stress Mitigation

Abiotic stressors such as drought, salinity, extreme temperatures, and heavy metal toxicity are a great threat to global food security [37,38]. Nanoparticles mitigate these effects through multiple physiological, biochemical, and molecular pathways, including regulation of phytohormones, enhancement of antioxidant defenses, and modulation of stress-responsive gene expression [29]. Their distinctive characteristics, which include a high ratio of surface to volume and quantum effects, allow them to interrelate with plant systems at a cellular and subcellular level and consequently initiate a cascade of favorable responses that strengthen plant resilience. As comprehensively reviewed by Zhou et al. [39], nanoparticles function as promising tools against environmental stress through multiple interconnected mechanisms (established). Their analysis emphasizes that the efficacy of nanoparticles depends critically on type, size, concentration, and application method—a theme that runs throughout the current review. The authors particularly highlight the role of silicon, zinc oxide, and titanium dioxide nanoparticles in enhancing photosynthetic efficiency, antioxidant defense, and osmotic regulation under drought, salinity, and extreme temperature conditions. This communication promotes better nutrient absorption, higher photosynthetic efficiency, and stress signaling pathway regulation, which eventually leads to an increase in stress resistance and agricultural productivity [19,40] (postulated), although the casual chain for NP application to yield improvement has not been fully traced in most studies. 

### 3.1. An Integrated Mechanistic Framework

The processes by which NPs enhance abiotic stress tolerance are not isolated but function as an integrated network. ROS balance serves as a central hub: mild, NP-induced ROS elevation activates antioxidant enzymes (SOD, CAT, APX) and stress signaling cascades, which in turn modulate phytohormone levels (ABA, SA, JA) and downstream gene expression (established). These hormonal changes regulate stomatal aperture, osmolyte accumulation, and stress-responsive transcription factors, ultimately coordinating plant adaptation. This integration explains why NPs can simultaneously improve drought tolerance, salinity resistance, and heavy metal detoxification—they target shared regulatory nodes rather than individual stress responses [41,42] (postulated). However, direct evidence showing that a single NP simultaneously engages all these pathways (ROS, hormonal, transcriptional) in the same plant under combined stress remains limited.

### 3.2. Distinguishing Nanoparticle Priming from Toxic Stress

A critical distinction must be made between nanoparticle-induced priming and nanoparticle-induced toxicity, as both can influence plant stress responses but through fundamentally different mechanisms. Priming refers to a preconditioning state induced by low, subtoxic doses of nanoparticles, wherein plants exhibit enhanced activation of defense responses upon subsequent stress exposure without sustaining significant growth penalties. In contrast, toxic stress occurs at high nanoparticle concentrations, leading to oxidative damage, growth inhibition, and cellular dysfunction. Table 2 summarizes key differences between these two phenomena.

### 3.3. Regulation of Phytohormone Levels

**Figure 2:**
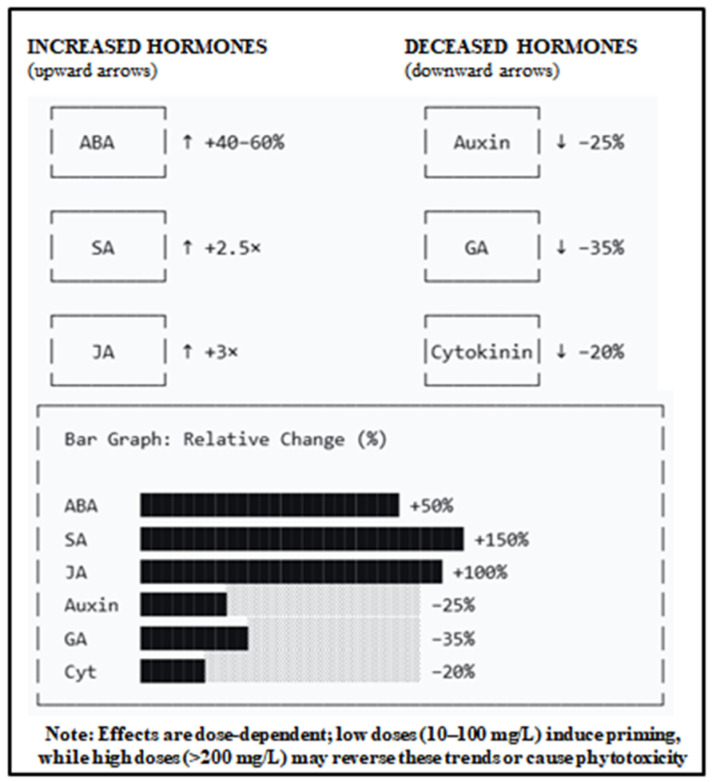
Nanoparticle-mediated regulation of phytohormone levels. Green upward arrows (↑) indicate increased hormone concentrations; red downward arrows (↓) indicate decreased concentrations. Quantitative fold-changes and percentage changes are shown where available from field-validated studies. Abscisic acid (ABA), salicylic acid (SA), and jasmonic acid (JA) are typically upregulated by low to moderate NP doses (10–100 mg/L), enhancing stress tolerance. Auxin, gibberellins (GA), and cytokinins are typically downregulated, reflecting a shift from growth to defense. The bar graph shows relative changes (%). Effects are dose-dependent; supraoptimal NP doses (>200 mg/L for ZnONPs, >50 mg/L for AgNPs) may cause phytotoxicity and disrupt normal hormone balance.

Phytohormones are important in the regulation of growth, development, and stress of plants (Figure 2). The biosynthesis, signaling, and transport of these important molecules can be greatly modulated by NPs, which improves the adaptation of plants to abiotic stresses [3]. The effects are NP-type and concentration-dependent: SiNPs elevate ABA and JA under salinity, while AgNPs alter ethylene and gibberellin levels in rice [14]. ZnO NPs combined with strigolactone mitigate cadmium toxicity in tomato by enhancing pigment synthesis and photosynthesis [43]. The synergistic effect of NPs and the phytohormones has also been reported in the case of combined usage of iron oxide nanoparticles (Fe_2_O_3_ NPs) and salicylic acid, which led to the growth and production of ajowan plants in salty conditions [43].

NPs influence the balance among ABA (drought/salinity), SA (pathogen defense), and JA (herbivory/necrotrophs), enabling coordinated responses to multiple stresses [19]. This postulate is supported by correlative evidence showing simultaneous changes in multiple hormone levels after NP treatment, but direct casual evidene (e.g., using hormone biosynthesis mutants) is currently lacking. SiNPs elevate auxin and ABA while suppressing cytokinin and gibberellin in leaves, indicating tissue-specific regulation [44]. Mesoporous silica NPs designed to release ABA enhance drought resistance in Arabidopsis via AtGALK2 gene regulation [14]. Silver NPs increase ABA levels, promoting stomatal closure and reducing water loss under drought [19]. However, the precise molecular interactions between NPs and specific hormone receptors remain largely unexplored [45] and represent a hypothesis requiring investigation using techniques such as surface plasmon resonance or molecular docking studies. 

Recent advances have examined the specific molecular pathways through which ABA, in combination with nanoparticle or nanoliposome delivery systems, regulates gene expression and cellular stress responses. Kutasy et al. [46] demonstrated that nanoliposome-formulated garlic extracts containing high ABA concentrations (up to 81 µg g^−1^) significantly upregulated stress-responsive genes in field-grown pea (*Pisum sativum*). The ABA signaling genes SnRK2.3 and ABF showed marked upregulation, activating the core ABA signaling cascade. Pathogenesis-related (PR) genes—PR1, PR2, PR4, and PR5—were overexpressed, demonstrating crosstalk between ABA signaling and biotic defense pathways. This upregulation was accompanied by enhanced glutathione metabolism and a 14.3% increase in thiamine (vitamin B1) content, which itself acts as a priming agent for systemic acquired resistance [46]. Such nanoparticle-enabled ABA delivery represents a mechanistically grounded strategy for inducing cross-tolerance against combined stresses.

### 3.4. Enhancement of Antioxidant Defense Systems

Abiotic stress cause oxidative stress through excessive ROX production, damaging cellular components. NPs counteract this by enhancing both enzymatic (SOD, CAT, APX, GPX, GR) and non-enzymatic, which would not only eliminate this harmful reactive oxygen species but would also restore the homeostasis of the cell [1,29]. Low doses of TiO_2_NPs upregulate SOD and CAT, scavenging ROS and protecting photosynthesis; higher doses overwhelm defenses, causing oxidative damage [19]. Cerium oxide, manganese (Mn_3_O_4_), and iron oxide (Fe_3_O_4_) nanoparticles exhibit intrinsic ROS-scavenging activity (mimicking SOD and catalase), directly neutralizing superoxide and H_2_O_2_ [47]. AgNPs enhance proline, flavonoids, and phenolics in pearl millet, demonstrating broad antioxidant system activation [30]. Iron oxide (Fe_3_O_4_) and cerium oxide NPs activate antioxidant enzymes while inhibiting cadmium accumulation in plants [48]. The threshold between beneficial ROS priming and toxic oxidative stress varies with NP type, size, and concentration—a critical consideration for application [21].

### 3.5. Improvement of Physiological Processes

**Figure 3:**
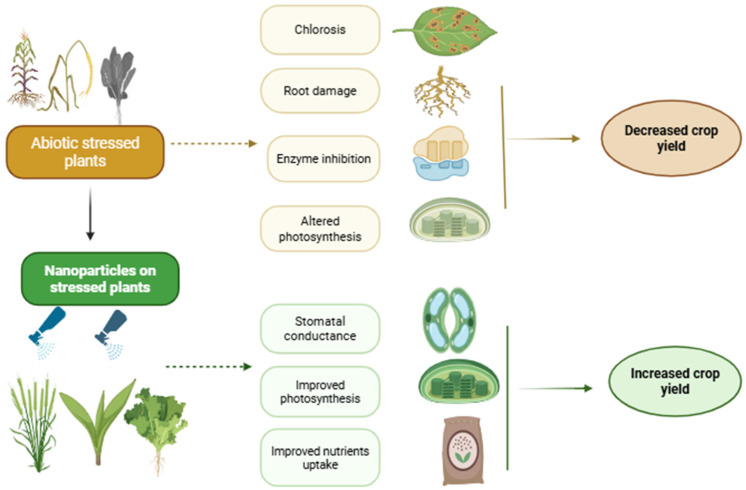
Nanoparticle application mitigates abiotic stress by improving photosynthesis, nutrient uptake, and stomatal conductance, thereby reducing stress damage and increasing crop yield.

Nanoparticles have a great impact in enhancing several physiological functions in plants, which result in increased growth and stress resistance (Figure 3). These improvements can be direct (NP-mediated enhancement of electron transport, Rubisco activation) or indirect (reduced oxidative damage preserving chloroplast function). ZnO NPs improve chickpea growth and biomass more effectively compared to the conventional use of ZnSO_4_ [1]. SiNPs boost the growth and yield of maize under drought conditions by manipulating the photosynthetic parameters, water use efficiency, and nutrient uptake [19]. Furthermore, it has also been noted that the application of iron oxide (Fe_2_O_3_) and titanium oxide NPs promote the metabolic and physiological processes of plants under drought conditions by increasing the water absorption and root hydraulic conductance of plants, which are more prone to use water efficiently [4]. Fe_2_O_3_ NPs increase net photosynthetic rate in maize and tomato by enhancing chlorophyll content and electron transport [37]. The magnitude of physiological improvement depends on NP concentration, with hermetic effects commonly observed low doses stimulate, high doses inhibit [49,50].

### 3.6. Type, Size, and Concentration-Dependent Effects

A recurring theme across all mechanisms is that NP effects are not generalizable which depend on physicochemical properties and application parameters. The key dependencies influencing NP efficacy and safety are summarized in Table 3. These dependencies must be considered when interpreting published results and designing applications. A concentration that is protective in one species or under one stress may be ineffective or harmful in another.

### 3.7. Iron Oxide Nanoparticle Mechanisms and Applications

Iron oxide nanoparticles ((Fe_3_O_4_, magnetite; γ-Fe_2_O_3_, maghemite) deserve particular attention due to their unique properties and agricultural potential. Unlike many metal NPs that primarily act as stressors, iron oxide NPs can function as both nanofertilizers (delivering essential iron) and signaling modulators with intrinsic ROS-regulating activity. Iron oxide NPs exhibit catalase-mimetic activity, directly scavenging H_2_O_2_ and reducing oxidative stress [48]. They also release Fe^2+^/Fe^3+^ ions, which are essential cofactors for antioxidant enzymes (SOD, CAT) and photosynthetic electron transport components. Also, they upregulate iron transporter genes (IRT1, FRO2), enhancing iron acquisition under deficiency [52]. Under drought, Fe_3_O_4_ NPs (50–100 mg/L) increase chlorophyll content, Fv/Fm, and water-use efficiency in wheat and maize while reducing H_2_O_2_ and MDA levels [4,53]. Under salinity, they improve K^+^/Na^+^ ratios and maintain photosynthetic activity. Under heavy metal stress (Cd, Pb), iron oxide NPs reduce metal uptake by competing for transport sites and promoting iron plaque formation on roots [52]. Low to moderate concentrations (20–100 mg/L) are generally beneficial; higher concentrations (>200 mg/L) can cause oxidative stress and growth inhibition due to excessive iron accumulation. The balance between beneficial iron nutrition and potential toxicity is narrow, requiring careful optimization [19].

### 3.8. Interaction with Soil Properties and Remediation of Abiotic Soil Stresses

Nanoparticles NPs influence soil physicochemical properties—water retention, nutrient availability, and microbial activity—which indirectly affect plant stress tolerance [54]. Some NPs (metal and metal oxide) have demonstrated that they can be used to clean up contaminated soils by immobilizing heavy metals or altering their degradability, thus lowering their bioavailability and reducing their toxicity to plants [15,55]. However, long-term ecological consequences—soil accumulation, microbial community shifts, nutrient cycling disruption—require rigorous study [41,50]. Future research must prioritize biodegradable nanoparticles, optimized application protocols, and integrated risk assessment frameworks to ensure environmental sustainability [56,57]. To provide a comprehensive overview of reported effects across different nanoparticle types, concentrations, and stress conditions, Table 4 summarizes representative studies on nanoparticle-induced abiotic stress mitigation in various crop species.

## 4. Mechanisms of Nanoparticle-Induced Biotic Stress Mitigation

Nanoparticles mitigate biotic stress in plants through two fundamentally distinct mechanisms: (1) direct antimicrobial activity, wherein nanoparticles physically or chemically damage pathogens without requiring active plant metabolism; and (2) induced resistance, wherein nanoparticles trigger the plant’s innate immune system to mount defense responses through systemic signaling pathways [71]. Understanding this separation is critical for designing nanoparticle-based crop protection strategies. Direct antimicrobial effects offer immediate, pathogen-targeted control, whereas induced resistance provides broader, longer-lasting protection but requires activation of plant signaling networks and carries a lower risk of pathogen resistance development. Table 5 summarizes the key differences between these two mechanisms.

### 4.1. Direct Antimicrobial Mechanisms

Direct antimicrobial activity refers to the ability of nanoparticles to kill or suppress plant pathogens through physical or chemical interactions that function independently of plant metabolism. This mechanism is particularly valuable for immediate pathogen control on leaf surfaces, in wounds, or during seed treatment where rapid intervention is needed.

#### 4.1.1. Membrane Disruption and Cell Wall Damage

Metal and metal oxide nanoparticles—particularly silver (AgNPs), copper oxide (CuONPs), and zinc oxide (ZnONPs)—bind to pathogen cell membranes via electrostatic interactions due to their surface charge. This binding leads to membrane depolarization, increased permeability, leakage of intracellular contents, and eventual lysis. For example, AgNPs penetrate fungal hyphae of *Fusarium oxysporum*, causing cytoplasmic shrinkage, vacuolation, and complete disruption of membrane integrity [71]. Scanning electron microscopy confirms that CuONPs induce severe morphological alterations in hyphae, including wrinkling, collapse, bending, and fragmentation [35]. Similarly, ZnONPs damage the cell walls of *Alternaria alternata* and *Botrytis cinerea*, leading to loss of cellular compartmentalization [72]. Iron oxide NPs (Fe_3_O_4_, γ-Fe_2_O_3_) exhibit moderate antimicrobial activity through membrane disruption and ROS generation, although this has been proposed based on studies with bulk iron salts and NP characterization data; direct visualization of membrane disruption by FeO_3_O_4_NPs in fungal cells has not been reported [73,74].

#### 4.1.2. Reactive Oxygen Species (ROS) Generation

Many nanoparticles catalyze the production of ROS—including superoxide anion (O_2_^−^), hydrogen peroxide (H_2_O_2_), and hydroxyl radicals (•OH)—directly on pathogen surfaces or within pathogen cells following internalization [75]. These ROS oxidize lipids, denature proteins, and fragment DNA, culminating in oxidative cell death. Copper oxide nanoparticles generate substantial ROS that disrupt *Fusarium solani* cellular components, reducing root rot disease incidence in cucumber by up to 83% compared to untreated controls [76]. Silver-chitosan nanoparticles inhibit *F. oxysporum* mycelial growth at concentrations up to 2000 ppm without harming tomato seedlings, with ROS-mediated damage confirmed by increased malondialdehyde levels in treated fungal cells [77]. Titanium dioxide nanoparticles (TiO_2_NPs), upon UV activation, produce hydroxyl radicals that effectively kill bacterial pathogens such as *Pseudomonas syringae* [78].

#### 4.1.3. Metal Ion Release and Enzyme Inhibition

Soluble metal ions released from nanoparticles (e.g., Cu^2+^, Ag^+^, Zn^2+^) can penetrate pathogen cells and exert multiple toxic effects. These ions inhibit essential metalloenzymes by displacing native cofactors, interfere with electron transport chains in mitochondria, generate additional ROS via Fenton-type reactions, and bind directly to DNA, causing replication errors. Copper oxide nanoparticles release Cu^2+^ ions that inhibit fungal cytochrome c oxidase and superoxide dismutase, achieving antifungal effects comparable to conventional fungicides (e.g., bavistin) at significantly lower concentrations [72]. The slow, sustained release of ions from nanoparticles provides prolonged protection compared to bulk metal salts.

#### 4.1.4. Physical Interference with Pathogen Structures

**Figure 4:**
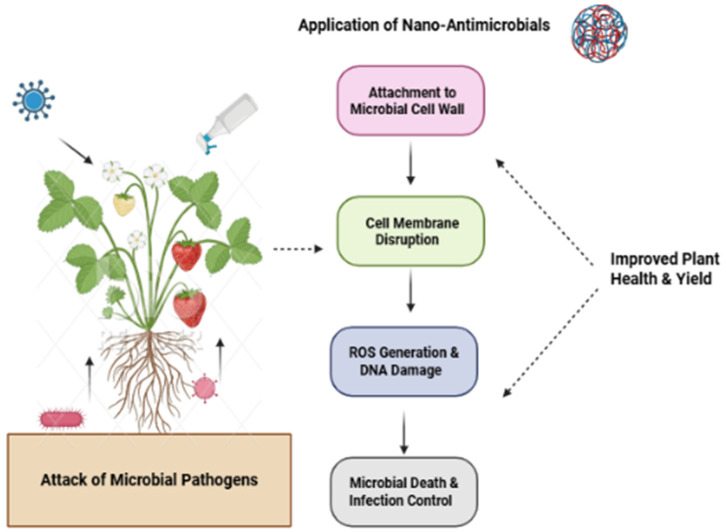
Role of nano-antimicrobials in controlling microbial pathogens and enhancing plant health.

Carbon nanotubes (CNTs) and silica nanoparticles (SiNPs) can physically adhere to pathogen surfaces, blocking nutrient uptake, impairing spore germination, or mechanically disrupting cell wall integrity. Multi-walled carbon nanotubes have been shown to entangle fungal hyphae, preventing normal growth and sporulation [79,80]. Silicon nanoparticles form a physical barrier on leaf surfaces when applied as foliar sprays, reducing fungal spore adhesion and penetration without directly killing the pathogen [25]. This physical mode of action is particularly valuable because it does not rely on toxic chemistry and poses a low risk of resistance development. The integrated role of nanoparticles in directly attacking microbial pathogens and simultaneously enhancing plant immune response are summarized in Figure 4.

### 4.2. Induced Resistance Mechanisms (SAR and ISR)

Unlike direct antimicrobial activity, induced resistance involves the activation of the plant’s own defense systems by nanoparticles, which act as elicitors or priming agents. This mechanism does not directly kill pathogens but prepares the plant to respond more rapidly and robustly upon subsequent pathogen attack. Two major forms of induced resistance are recognized in plant pathology:

- Systemic Acquired Resistance (SAR): A salicylic acid (SA)-dependent pathway that confers long-lasting, broad-spectrum resistance against a wide range of pathogens, typically following a localized infection or elicitor treatment.
- Induced Systemic Resistance (ISR): Typically jasmonic acid (JA) and ethylene (ET)-dependent, often triggered by beneficial rhizobacteria or specific elicitors, and effective primarily against necrotrophic pathogens and herbivores.

Nanoparticles can activate one or both pathways depending on their physicochemical properties, concentration, and application method.

#### 4.2.1. Activation of Pathogenesis-Related (PR) Genes

A hallmark of induced resistance is the upregulation of pathogenesis-related (PR) genes, which encode proteins with direct antimicrobial activity (e.g., chitinases, glucanases, thaumatin-like proteins) or regulatory functions. Nanoparticles trigger PR gene expression through the SA and JA signaling cascades. Silicon nanoparticles induce the expression of PR1 (unknown function, marker for SAR), PR2 (β-1,3-glucanase), and PR5 (thaumatin-like protein) in rice, enhancing resistance against *Magnaporthe oryzae* (blast disease) by degrading fungal cell walls and disrupting membrane integrity [81,82]. Copper oxide nanoparticles increase the activity of chitinase (PR3) and β-1,3-glucanase (PR2) in tomato, enzymes that hydrolyze the major structural components of fungal cell walls [83]. Iron oxide NPs (Fe_2_O_3_) have been shown to induce PR gene expression and enhance resistance against bacterial pathogens in tomato and tobacco [84]. Recent work using nanoliposome-formulated garlic extracts—containing high abscisic acid (ABA) concentrations (up to 81 µg g^−1^)—demonstrated significant upregulation of PR1, PR2, PR4, and PR5 in field-grown pea plants (*Pisum sativum*), alongside enhanced thiamine metabolism and glutathione transferase activity [46]. This study provides direct molecular evidence that nanoparticle-enabled delivery of phytohormones can activate coordinated defense gene expression.

#### 4.2.2. Salicylic Acid (SA) and Systemic Acquired Resistance (SAR)

Several nanoparticles elevate endogenous SA levels, the primary signaling molecule for SAR. SA accumulation triggers the redox-regulated oligomerization of NPR1 (nonexpressor of PR genes), which then translocates to the nucleus and activates the transcription of PR genes. SA also inhibits catalase activity, leading to elevated H_2_O_2_ levels that further reinforce defense signaling. Chitosan nanoparticles increase SA content and induce SAR in tomato against *Botrytis cinerea* (gray mold), resulting in reduced lesion size and disease severity [85]. Silver nanoparticles trigger SA accumulation in *Nicotiana benthamiana*, leading to enhanced resistance against *Pseudomonas syringae* without direct antibacterial activity at the applied concentration [86]. Iron oxide NPs have been reported to activate SA-dependent defense responses in several crops [87]. SAR provides long-lasting, broad-spectrum resistance and is particularly effective against biotrophic and hemibiotrophic pathogens.

#### 4.2.3. Jasmonic Acid (JA) and Ethylene (ET) Pathways in ISR

Other nanoparticles primarily activate JA/ET signaling pathways characteristic of ISR. This pathway is typically induced by necrotrophic pathogens and herbivorous insects and involves the activation of defense genes such as lipoxygenase (LOX), phenylalanine ammonia-lyase (PAL), and proteinase inhibitors.

Zinc oxide nanoparticles increase JA levels in chickpea, leading to enhanced defense against *Fusarium wilt* with up to 90% disease reduction compared to untreated controls [20]. Copper oxide nanoparticles activate both JA and ET signaling in watermelon, reducing *Fusarium wilt* severity and bacterial fruit blotch incidence [88]. The JA pathway also triggers the production of phytoalexins—antimicrobial secondary metabolites—and reinforces cell walls through lignin deposition.

#### 4.2.4. Defense Priming: A Key Feature of Nanoparticle-Induced Resistance

A critical concept in induced resistance is defense priming—the phenomenon where treated plants exhibit faster, stronger, and more sustained activation of defense responses upon subsequent pathogen challenge, without constitutive expression of defense genes under non-stress conditions. Priming avoids the growth-defense trade-off, allowing plants to allocate resources to growth when pathogens are absent while maintaining enhanced protection when needed. Nanoparticles are particularly effective priming agents. Plants pretreated with low, subtoxic doses of AgNPs show minimal changes in basal defense gene expression but, upon pathogen infection, exhibit rapid and amplified PR gene expression, ROS burst, and callose deposition [88]. This priming effect is often mediated by epigenetic modifications, including histone acetylation at defense gene promoters and changes in DNA methylation patterns, which persist for days to weeks after nanoparticle treatment [89]. Silicon nanoparticles prime wheat plants for enhanced chitinase and glucanase activity specifically upon *Fusarium* infection, without elevating these enzymes in healthy plants [25].

#### 4.2.5. Synergy Between Direct Antimicrobial and Induced Resistance Mechanisms

**Figure 5:**
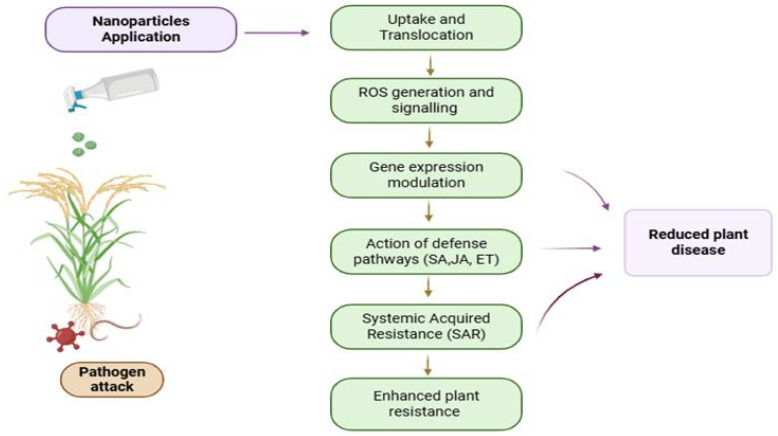
Mechanisms of nanoparticles in mitigating biotic stress in plants.

In many agricultural applications, nanoparticles function through both direct antimicrobial activity and induced resistance simultaneously, providing a dual layer of protection. For example, copper nanoparticles directly kill *Fusarium* hyphae via membrane disruption and ROS generation while also upregulating PR genes and enhancing SA-dependent SAR in the host plant [83]. This synergy reduces the required dose of active ingredients, minimizes the risk of pathogen resistance, and provides more robust and durable disease control than either mechanism alone. Iron oxide NPs, though less potent as direct biocides, effectively prime plant defenses and serve as carriers for antifungal agents [87]. Understanding the relative contribution of each mechanism for a given nanoparticle–pathogen–crop combination is essential for optimizing application strategies. A visual summary of the overall mechanism by which nanoparticles mitigate biotic stress in plants is present in Figure 5.

### 4.3. Nanoparticles as Carriers for Biocontrol Agents

Nanoparticles intrinsic direct antimicrobial and resistance-inducing properties, which can serve as carriers or delivery vehicles for biocontrol agents, enhancing stability, bioavailability, and targeted delivery. This function is distinct from direct antimicrobial or resistance-inducing activities.

#### 4.3.1. Delivery of Biocontrol Microorganisms

NPs can encapsulate and protect beneficial microorganisms (e.g., Trichoderma, Bacillus) from UV degradation, desiccation, and microbial competition. Chitosan NPs have been used to deliver *Bacillus subtilis* lipopeptides, enhancing antifungal activity against *Rhizoctonia solani* [57]. Silica NPs improve the persistence of biocontrol agents in the phyllosphere and rhizosphere.

#### 4.3.2. Delivery of Botanical Extracts and Natural Products

Nanoliposome formulations of garlic extracts (rich in ABA and sulfur compounds) improved bioavailability, enabling field-level control of *Fusarium oxysporum f.* sp. *pisi* in pea [46]. CuONPs loaded with chitosan deliver antifungal compounds directly to infection sites, protecting banana, tea, and citrus against powdery mildew and rust [90].

#### 4.3.3. Controlled Released and Target Delivery

Smart delivery systems enable stimuli-responsive release—NPs release cargo in response to pathogen enzymes (e.g., fungal cell wall-degrading enzymes), pH changes, or other environmental cues. This approach reduces off-target effects and improves efficacy [91].

#### 4.3.4. Iron Oxide NPs as Carriers

Iron oxide NPs (Fe_3_O_4_) have emerged as promising carriers due to their biocompatibility, magnetic properties enabling targeted delivery, and ability to be functionalized with antifungal compounds. They have been used to deliver antifungal peptides and essential oils against *Fusarium* and *Botrytis species* [87]. To provide a comprehensive overview of experimental evidence across different nanoparticle types, target pathogens, and host plants, Table 6 compiles representative studies on nanoparticle-mediated biotic stress mitigation, including concentrations, causal organisms, and observed effects.

## 5. Cross-Tolerance: Concurrent Mitigation of Biotic and Abiotic Stresses

Among the major benefits of the use of nanoparticles (NP) in agriculture is the ability to trigger cross-tolerance, where plants become resistant to both biotic and abiotic stresses simultaneously [106]. In natural field environment, crops are hardly subjected to one stress factor, but they are commonly exposed to complex mixtures of environmental limitations, including drought, salinity, and toxicity of heavy metals, as well as pathogen attack, simultaneously. NPs act as stress-priming agents that stimulate core defense networks, enabling plants to be responsive and effective to various stressors [107]. This wide-range tolerance is caused by the stimulation of shared signaling pathways, an increase in cellular homeostasis, and modulation of molecular processes that control plant growth and defense. However, a critical distinction must be made when interpreting physiological improvements attributed to nanoparticles: direct effects (where nanoparticles actively enhance physiological processes such as photosynthetic electron transport, nutrient ion uptake, or stomatal regulation) versus indirect effects (where physiological improvements result secondarily from reduced stress damage, e.g., less oxidative damage to chloroplasts leading to better photosynthesis).

### 5.1. Direct vs. Indirect Physiological Effects of Nanoparticles

Cross-tolerance emerges from the convergence of three interconnected networks: ROS signaling, hormonal crosstalk, and transcriptional reprogramming.

ROS signaling as a central hub: Both biotic and abiotic stresses trigger ROS production. At low, controlled levels, ROS act as signaling molecules activating antioxidant enzymes and stress-responsive transcription factors. Nanoparticles modulate this balance: low doses induce controlled ROS elevation (priming), while high doses cause oxidative damage. This biphasic response is the mechanistic basis for hormesis [108].

Hormonal crosstalk: NPs influence the balance among ABA (drought/salinity), SA (pathogen defense), and JA (herbivory/necrotrophs). ABA-SA-JA crosstalk enables coordinated responses: ABA-induced stomatal closure reduces water loss but may increase susceptibility to certain pathogens; SA and JA pathways can be antagonistic or synergistic depending on stress combinations. NPs that simultaneously modulate multiple hormones (e.g., SiNPs, ZnONPs, iron oxide NPs) are particularly effective at inducing cross-tolerance [19,109].

Transcriptional reprogramming: Downstream of ROS and hormonal signals, NPs activate stress-responsive transcription factors (e.g., DREB, NAC, WRKY, MYB) and defense genes. This reprogramming enables plants to mount rapid, coordinated responses to subsequent challenges [110].

Integrated across scales: This framework connects molecular events (ROS, hormones, transcription) to cellular outcomes (antioxidant activation, osmolyte accumulation) to whole-plant responses (stomatal regulation, growth-defense balance) to agronomic outcomes (yield, stress tolerance). Nanoparticles that target multiple nodes in this network—such as iron oxide NPs, which modulate ROS directly (catalase-mimetic) and indirectly (antioxidant gene induction)—offer unique advantages for cross-tolerance induction.

Table 7 distinguishes direct and indirect effects of nanoparticles on key physiological processes.

Understanding this separation is essential for rational design of nanoparticle applications. For example, if a nanoparticle improves photosynthesis primarily by reducing oxidative damage (indirect effect), the same benefit might be achieved with conventional antioxidants. Conversely, if a nanoparticle directly enhances electron transport (direct effect), it offers a unique value proposition

### 5.2. Mechanistic Analysis of Photosynthetic Enhancement

Photosynthesis is often reported to be “improved” by nanoparticles under stress, but the specific parameters and mechanisms vary considerably. Below we detail the key photosynthetic measurements, their physiological meanings, and nanoparticle effects with quantitative examples.

#### 5.2.1. Photosynthetic Parameters and Their Interpretation

The key photosynthetic parameters commonly used to assess nanoparticle effects on plant stress tolerance, along with their physiological meanings and typical responses, are summarized in Table 8.

#### 5.2.2. Nanoparticle Effects on Photosynthetic Parameters: Quantitative Evidence

Silicon nanoparticles (SiNPs) in drought-stressed wheat: Fv/Fm increased from 0.68 (drought control) to 0.79 (SiNP-treated), ΦPSII increased by 38%, and Pn increased from 12.4 to 19.7 µmol CO_2_ m^−2^ s^−1^. These improvements were attributed to both direct effects (SiNPs upregulating *PsbA* and *PsbD* genes encoding D1 and D2 proteins of PSII) and indirect effects (reduced lipid peroxidation in thylakoid membranes) [19,53].

Titanium dioxide nanoparticles (TiO_2_NPs) in salinity-stressed pea: Fv/Fm recovered from 0.71 to 0.83, NPQ decreased by 32% (indicating reduced energy dissipation), and Pn increased by 45% compared to salt-stressed controls. The primary mechanism was direct: TiO_2_NPs enhanced the activity of Rubisco activase and increased chlorophyll *a* and *b* content by 28% and 35%, respectively [62].

Zinc oxide nanoparticles (ZnONPs) in cadmium-stressed tomato: Chlorophyll fluorescence imaging revealed that ZnONPs prevented the stress-induced decrease in ΦPSII (from 0.52 to 0.74) and maintained qP near control levels. However, at concentrations above 200 mg/L, ZnONPs caused phytotoxic effects: Fv/Fm dropped to 0.58, NPQ increased by 60%, and visible chlorosis appeared [51]. This highlights the critical importance of dose optimization.

#### 5.2.3. Dose-Dependent Effects and Hormesis in Photosynthesis

Nanoparticles exhibit a biphasic dose response known as hormesis—low doses stimulate physiological processes, while high doses inhibit or damage them. Table 9 summarizes hormetic and phytotoxic concentration ranges for different NP types on photosynthetic parameters.

The hormetic effect is mechanistically explained by low-dose ROS signaling: at subtoxic concentrations, nanoparticles generate mild oxidative stress that activates antioxidant defenses and stress acclimation pathways, including upregulation of photosynthetic genes. At high concentrations, ROS production overwhelms cellular defenses, causing oxidative damage to PSII reaction centers, D1 protein degradation, and chlorophyll destruction. This biphasic response must be carefully considered in agronomic applications.

### 5.3. Mechanistic Analysis of Nutrient Uptake Enhancement

Nanoparticles can improve plant nutrient uptake through two distinct mechanisms that must be separated: (1) nanoparticles as nutrient carriers (direct delivery) and (2) nanoparticles as physiological regulators (indirect enhancement of uptake capacity).

#### 5.3.1. Nanoparticles as Nutrient Carriers (Direct Mechanism)

When nanoparticles contain essential nutrients (e.g., ZnONPs for zinc, CuONPs for copper, Fe_3_O_4_NPs for iron), they can serve as nanofertilizers, delivering bound nutrients directly to plant tissues. This mechanism offers several advantages over conventional salts: controlled release (slow dissolution provides sustained availability), reduced soil fixation (less prone to precipitation with phosphates), enhanced penetration (small size allows entry through apoplastic pathways), and improved bioavailability. Quantitatively, ZnONPs at 100 mg/L increased grain zinc content in wheat from 18.5 ppm (control) to 42.3 ppm under saline conditions, compared to 26.1 ppm with ZnSO_4_, demonstrating superior biofortification efficiency [59,111].

#### 5.3.2. Nanoparticles as Physiological Regulators (Indirect Mechanism)

Independently of nutrient content, nanoparticles upregulate ion transporter genes, enhancing the plant’s intrinsic capacity to acquire nutrients from the rhizosphere. This mechanism is particularly valuable for nutrients not supplied by the nanoparticles themselves. Key transporter genes affected by nanoparticles include: Iron-Regulated Transporter 1 (IRT1), upregulated 2–4 fold by SiNPs; NRT1.1 and NRT2.1 (nitrate transporters), upregulated by TiO_2_NPs; PHT1 (phosphate transporter), upregulated by CeO_2_NPs; and KT/HAK/KUP (potassium transporters), upregulated by ZnONPs in tomato under salinity, improving K^+^/Na^+^ selectivity [51]. Bioavailability is critical: even with transporter upregulation, nutrient uptake is limited by actual nutrient availability in the growth medium.

#### 5.3.3. Distinguishing Carrier vs. Regulatory Effects: Experimental Approaches

To determine whether a nanoparticle improves nutrient uptake as a carrier or physiological regulator, researchers can use isotopic labeling (e.g., ^65^Zn-labeled ZnONPs), nutrient-free controls, transporter mutant lines (e.g., *irt1* knockout), and comparative dissolution studies.

### 5.4. Mechanistic Analysis of Water Utilization Efficiency

Water use efficiency (WUE)—typically defined as biomass produced per unit water transpired (WUEₑcₒ) or CO_2_ assimilated per unit water lost (WUEᵢₙₜ)—is often reported to be “improved” by nanoparticles under drought stress. However, the underlying mechanisms require detailed analysis of stomatal regulation, hormonal signaling (especially ABA), aquaporin activity, and root hydraulic conductance.

#### 5.4.1. Stomatal Regulation and ABA-Mediated Processes

Abscisic acid (ABA) is the master regulator of stomatal closure under water deficit. Nanoparticles can modulate WUE through ABA-dependent and ABA-independent pathways. ABA-dependent pathway: AgNPs (10–50 mg/L) increase endogenous ABA levels in wheat leaves by 40–60%, leading to stomatal closure, reduced transpiration rate (Tr decreased by 35%), and increased WUE (increased by 28%) without proportional reduction in photosynthesis [112]. This effect is direct—AgNPs upregulate *NCED3* (a key ABA biosynthesis gene) and *SnRK2.6* (an ABA signaling kinase). ABA-independent pathway: SiNPs (200 mg/L) reduce stomatal aperture and density through mechanisms not fully dependent on ABA from 0.32 to 0.18 mol H_2_O m^−2^ s^−1^ under drought, while maintaining photosynthesis, resulting in a 45% increase in intrinsic WUE (Pn/gs) [25]. Controversial effects: some NPs (TiO_2_NPs at low doses) increase stomatal conductance under mild drought, but improve WUE if photosynthetic gains outpace transpiration increases [63].

#### 5.4.2. Aquaporin Regulation

Aquaporins (AQPs) are water channel proteins that facilitate water movement across cell membranes. Nanoparticles can modulate AQP gene expression, affecting root water uptake and leaf hydraulic conductivity. SiNPs upregulate *PIP1;3* and *PIP2;2* in maize roots under drought, increasing root hydraulic conductivity (Lpr) by 55% and maintaining water uptake despite soil drying [19]. ZnONPs upregulate *TIP1;1* and *TIP2;1* in tomato leaves, facilitating vacuolar water storage and osmotic adjustment under salinity [51]. CuONPs downregulate *NIP5;1* in Arabidopsis at high concentrations (>100 mg/L), reducing boron uptake and causing growth inhibition—an example of phytotoxicity through AQP dysregulation.

#### 5.4.3. Root Hydraulic Conductance and Whole-Plant Water Relations

Root hydraulic conductance (Lpr) is a major determinant of plant water status. Nanoparticles can affect Lpr through Casparian strip modification (SiNPs induce suberin deposition), root architecture (ZnONPs increase root length density by 35% under drought), and xylem vessel integrity (low-dose CuONPs preserve xylem vessels; high doses cause collapse [76,111].

#### 5.4.4. Quantitative WUE Improvements: Examples and Mechanisms

Quantitative improvements in water use efficiency (WUE) achieved by different nanoparticle types under various stress conditions, along with their primary mechanisms of action, are compiled in Table 10.

### 5.5. Cross-Tolerance: Integration of Photosynthetic, Nutritional, and Water Relations

The preceding subsections have analyzed photosynthesis, nutrient uptake, and water utilization as separate processes. However, cross-tolerance emerges from their integration. Improved WUE (via stomatal regulation) reduces water stress, which in turn preserves photosynthetic electron transport (indirect effect on photosynthesis). Enhanced nutrient uptake (via transporter upregulation) provides essential cofactors for photosynthetic enzymes (e.g., Mg^2+^ for chlorophyll, Mn^2+^ for the oxygen-evolving complex), directly improving photosynthesis. Reduced oxidative damage (from improved antioxidant systems) protects both chloroplasts (preserving photosynthesis) and root membranes (maintaining nutrient and water uptake).

Nanoparticles that simultaneously target multiple processes—such as ZnONPs providing zinc (direct nutrient), upregulating *IRT1* (physiological regulation), and inducing ABA synthesis (stomatal regulation)—offer the greatest potential for inducing robust cross-tolerance. However, the dose must be carefully optimized to avoid the phytotoxic effects described in Section 5.3.3.

### 5.6. Examples of Nanoparticles Inducing Cross-Tolerance

Several studies have demonstrated the ability of specific NPs to induce cross-tolerance in various crops: these nanomaterials can be engineered to interact with precise cellular and molecular targets, leveraging their physicochemical properties to bolster innate defense mechanisms [113]. This includes improving nutrient uptake, enhancing plant defense pathways, and mitigating abiotic stress responses, which collectively contribute to the plant’s resilience against diverse environmental challenges [114,115].

#### 5.6.1. Selenium Nanoparticles (SeNPs)

**Figure 6:**
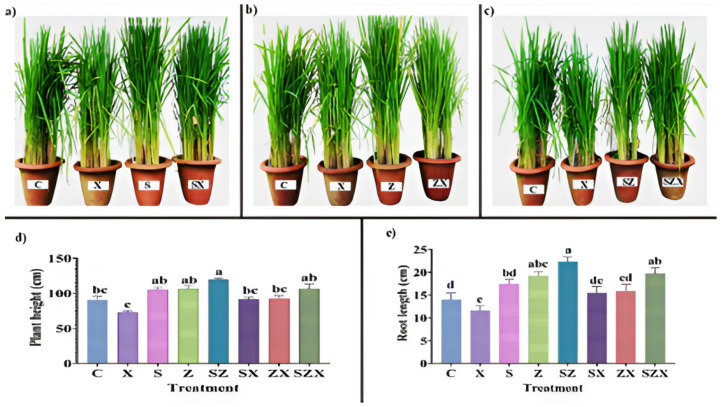
Salinity stress amelioration through selenium and zinc oxide nanoparticles in rice [adapted from [127]]. Different letters above the bars indicate statistically significant differences among treatments (*p* < 0.05).

SeNPs utilization is a promising approach to improving agricultural production through alleviating a broad spectrum of environmental stressors (Figure 6). In particular, SeNPs demonstrate superiority to other nanoparticles because of the critical role of selenium in the process of plant defense mechanism activation [116]. This is an essential trace element that is associated with resiliency to obstacles like heavy metals, salinity, and pathogens due to its antioxidant effects at the right proportions [117]. Thus, the interpretation of the specific uptake, translocation, and accumulation pathways of SeNPs in different plant species is the key to the optimal use and the most effective protection without the development of phytotoxic activity [118]. Since they are highly biocompatible and powerful, plant-mediated SeNPs are expected to transform the agricultural industry through the production of selenium-based antimicrobial and biofortifying products. It will require additional deep studies of the in vivo mechanisms used by plant-based SeNPs to counteract environmental stresses, which could be done through the combined work of plant physiologists, biochemists, molecular biologists, and nanotechnologists [41]. Additionally, nanobiotechnology, including the application of biosynthesized SeNPs, can be a sustainable and environmentally friendly method of improving agricultural resilience in terms of introducing a range of tools, including nano-pesticides and nano-fertilizers [119].

#### 5.6.2. Zinc Oxide Nanoparticles (ZnO NPs)

Zinc Oxide Nanoparticles have a potential future of improving the hardiness of plants facing a wide variety of environmental stressors (Figure 6). The nanoscale materials, which are commonly between 1 and 100 nm, utilise the fact that zinc is central to many physiological responses to enhance defence responses and growth rates of plants in unfavorable environments [120]. In particular, ZnO NPs have been proven to be effective biostimulants, which improve the growth and antioxidant activity of plants, antifungal, antibacterial, and nutritional, which increase yield, biomass, root development, and chlorophyll concentration [121]. In addition to these overt advantages, ZnO NPs used as seed primers have also been found to promote the alleviation of early stress signals through increased nutrient uptake and antioxidant capacity, which improves the effects of the environmental and biotic stressors at critical developmental phases [122]. The next generation of research efforts must involve understanding the molecular processes on which ZnO NP-mediated stress tolerance depends, especially when it comes to their engagement with the plant defense systems and the biomolecule targeting, to enable the creation of novel and stress-resistant breeds [123]. 

The exact mechanisms through which ZnO NPs regulate gene expression and protein synthesis may be further investigated to reveal further ZnO NP-based pathways to improve plant adaptive responses [82,124]. Moreover, ZnO NPs were found to regulate salt signaling pathways through phytohormones, which enhances photosynthetic rates and general salinity of plants in the presence of an osmotic stress [125]. This functionality can be explained by the fact that ZnO regulates the metabolism of phytohormones and different enzyme processes, including superoxide dismutase and dehydrogenases, which play an essential role in reducing stress [126]. Their ability to serve as nanofertilizers and nanopesticides and vehicles used in the delivery of plant growth regulators further increases their application in sustainable agriculture. Such multifunctional application makes ZnO NPs a key element in the creation of integrated approaches to crop improvement, as well as crop protection [111]. Research showed that treatment with Se-NPs and ZnO-NPs significantly alleviated salinity stress by enhancing the plant antioxidant defense system (Figure 6). Activities of superoxide dismutase (50.06%), catalase (59.92%), ascorbate peroxidase (104.28%), and peroxidase (85%) were markedly increased, leading to improved ROS scavenging and reduced lipid peroxidation. Combined nanoparticle application restored plant growth and physiological performance, with increases of 46.32% in plant height, 70.53% in root length, and 100.7% in grains per spike under salinity stress. Moreover, mineral accumulation in rice grains was enhanced, including Zn (31.8 ppm), Se (0.57 ppm), and Fe (7.4 ppm). These results demonstrate that green-synthesized Se-NPs and ZnO-NPs, particularly in combination, effectively improve salinity tolerance and contribute to rice biofortification [127].

The above (Figure 6), panel (a) shows photographs of rice plants at harvest. Plants treated with combined SeNPs and ZnONPs exhibit greener leaves, taller stature, and larger panicles compared to salinity-stressed controls, panel (b) shows close-up photographs of rice panicles (seed heads). Combined NP treatment resulted in longer panicles with more filled grains per spike compared to salinity-stressed plants, panel (c) shows photographs of rice grains. Combined NP treatment produced larger, more numerous, and better-filled grains compared to the shriveled, smaller grains observed under salinity stress alone, panel (d) is a bar graph showing antioxidant enzyme activities including superoxide dismutase (SOD), catalase (CAT), ascorbate peroxidase (APX), and peroxidase (POD). Values represent enzyme activity units per milligram of protein, and panel (e) is a bar graph showing plant growth parameters including plant height in centimeters, root length in centimeters, and grains per spike as a number. Values are means ± standard deviation. Also, the eode definitions indicates: C means control (non-stressed, no NP treatment). S means salinity stress only (no NP treatment). Z means zinc oxide nanoparticles (ZnONPs) alone at 50 mg/L. Se means selenium nanoparticles (SeNPs) alone at 25 mg/L. Z + Se means combined treatment (ZnONPs 50 mg/L + SeNPs 25 mg/L). The findings indicates that combined NP treatment (Z + Se) increased SOD activity by 50 percent, CAT by 60 percent, APX by 104 percent, and POD by 85 percent compared to salinity stress alone (S). Combined treatment increased plant height by 46 percent, root length by 71 percent, and grains per spike by 101 percent compared to salinity stress alone. Individual NP treatments (Z or Se alone) showed intermediate effects, while the combination (Z + Se) was most effective, indicating synergy.

#### 5.6.3. Carbon Nanotubes (CNTs)

The increasing world population and related environmental pressures of reduced arable agricultural land and climate change require sophisticated solutions to strengthen crop resilience to diverse stresses [79]. Climate change especially exacerbates abiotic stresses such as drought, salinity, and extreme temperatures, which result in significant crop yield and agricultural productivity decline [128]. Carbon Nanotubes are some of these nanomaterials that have shown great potential in diminishing the effects of both biotic and abiotic stress to plants. They possess attracting physiochemical characteristics, high surface area, excellent mechanical and thermal strength, which makes them good tools to use in agriculture [41,129]. These nano dimensions are associated with their distinct characteristics that are dissimilar to bulk carbon and graphite, enabling diverse chemical reactivation and interactions in the biological systems [130]. Different applications of the nanotubes are further broadened by the different classifications of single-walled nanotubes and multi-walled nanotubes based on their layered structure and diameter [131]. Carbon nanotubes with outer walls, such as 2–100 nm and inner walls 1–3 nm, have a better density, tensile strength, and electrical conductivity than those of single-walled carbon nanotubes and can be especially beneficial in biotechnological applications [80,132]. It has been demonstrated that the usage of CNTs can enhance the growth of roots and the uptake of nutrients by plants, which results in the overall improvement of the growth parameters, namely the number of roots, the length of roots, and the total length of plants [133].

#### 5.6.4. Iron Oxide Nanoparticles (Fe_3_O_4_/Fe_2_O_3_NPs)

Iron oxide NPs mitigate drought (increased chlorophyll, Fv/Fm), salinity (improved K^+^/Na^+^ ratios), and heavy metal stress (reduced Cd uptake) while inducing PR genes against bacterial pathogens. Their catalase-mimetic activity provides direct ROS scavenging, complementing antioxidant enzyme induction. The therapeutic window (20–100 mg/L) is narrow; doses >200 mg/L cause oxidative stress and iron toxicity [52,84,87].

#### 5.6.5. Silicon Nanoparticles (SiNPs)

In addition to mitigating the effects of abiotic stresses (e.g., salt tolerance), SiNPs are also capable of causing resistance against biotic ones such as blast disease in rice. This cross-tolerance is explained by the fact that SiNPs enhance the strength of cell walls, which physically becomes more resilient to the invasion of pathogens, as well as defense gene activation [25]. Additionally, SiNPs lead to greater plant vigor and general defenses, and lead to better stress tolerance and productivity. The improvement in plant resilience is made possible by a concerted action of physiological, metabolomic, and molecular reactions, which help plants to become more resilient to different environmental challenges [134]. In particular, it has been postulated that the development of a binary film on the epidermal cell wall through absorbed SiNPs would physically hinder fungal, bacterial, and nematodal infections [135]. Such a physical obstacle is also enhanced by the fact that SiNPs can accomplish the process of modulating gene expression and causing the upregulation of pathogenesis-related proteins and enzymes that detoxify reactive oxygen species, which contributes to the enhancement of the innate defense of the plant [136].

#### 5.6.6. Chitosan Nanoparticles

Chitosan nanoparticles have shown a promising future in improving the resilience of plants to the diverse environment, including living organisms and unfavorable environments [137]. It is a chitin-based biopolymer that is becoming widely known because of its versatile use in the protection of plants and stimulating their growth [123]. In particular, chitosan nanoparticles have been demonstrated to cause remarkable amounts of membrane stability index, chlorophyll contents, proline contents, carotenoids, and activities of different antioxidant enzymes, enhancing the plant defense mechanisms. In addition to these physiological improvements, chitosan nanoparticles also regulate gene transcription pathways related to stress responses and support physical defenses against the invasion of pathogens [138]. Such enhancement of innate immunity, as well as enhanced physiological and biochemical indicators, makes chitosan nanoparticles one of the promising approaches to sustainable agriculture [139]. Their effectiveness is due to their nanoscale characteristics, which enable them to find their way to cells better and translocate active compounds than bulk chitosan [140]. Chitosan nanoparticles have been noted to trigger the development of osmoprotectants in plants, which helps them to endure cold stress, and their use as seed priming agents can make plants immune to salinity stress [141].

Moreover, they are biocompatible, biodegradable, and have low allergenicity, which highlights their application as an environmentally friendly alternative to traditional agrochemicals [23,142]. These properties, combined with their ability to induce a range of physiological and biochemical events between the molecular and macroscopic scale, make them useful in sustainable agriculture to safeguard plants and promote plant growth [130]. Furthermore, chitosan nanoparticles can be combined with other biostimulants, which have synergistic effects and increase the effectiveness of each of them by enhancing bioavailability and controlled delivery of active molecules [143]. The high control of physicochemical characteristics, including size and surface charge, makes it possible to achieve specific applications that ensure optimal nutrient absorption, photosynthetic performance, and overall crop performance [144]. Chitosan, a linear polysaccharide cationic polysaccharide that is a polymer of β-joined N-acetyl-d-glucosamine, is also an elicitor that promotes the growth of plants and activates defense against pathogens [138]. Such elicitor activity can be explained by the fact that chitosan causes some physicochemical responses, such as lignification, changes in ion flux, cytoplasmic acidification, membrane depolarization, protein phosphorylation, and the action of chitinase and glucanase, all of which strengthen the self-defense system of the plant. The inherent ability to stimulate natural immune response is what makes chitosan an attractive alternative to conventional agrochemicals, as it is inherently more biocompatible, biodegradable, and cost-effective [145,146].

**Figure 7:**
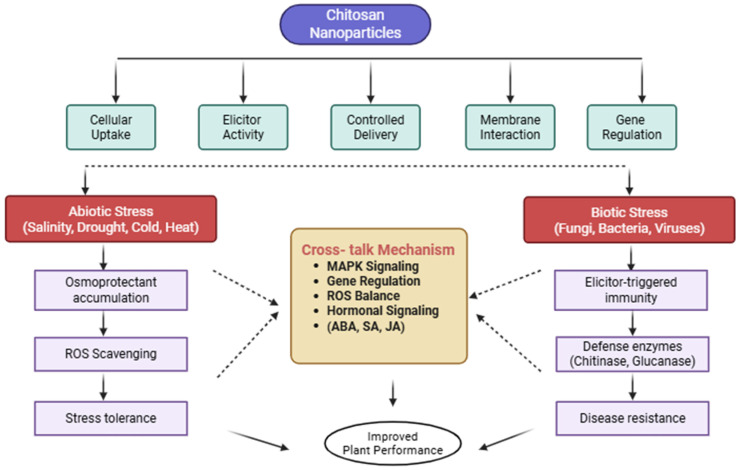
Cross-talk of biotic and abiotic stress mitigation by chitosan nanoparticles. Chitosan enhances plant resilience by regulating cellular uptake, gene expression, and defense responses, leading to improved tolerance to abiotic stress and resistance to pathogens.

The antimicrobial activity of chitosan nanoparticles is further increased by its polycationic character that gives them an advantage over traditional growth regulators in terms of being more environmentally sustainable than their bulk counterparts [147]. Researchers synthesized a novel engineered nanomaterial (Cs–Se NMs) via a Schiff base reaction between oxidized chitosan and selenocystamine hydrochloride to alleviate salt stress in plants. Application of 300 mg/L Cs–Se NMs significantly enhanced antioxidant enzyme activities in rice shoots, with superoxide dismutase, catalase, and peroxidase increasing by 3.19, 1.79, and 1.85 fold compared with the NaCl-treated group, while malondialdehyde content decreased by 63.9%. Transcriptomic analysis revealed upregulation of genes associated with oxidative stress responses and MAPK signaling pathways. In addition, Cs–Se NMs increased both the abundance and diversity of rhizobacteria and reshaped the microbial community structure. These findings highlight the potential of engineered nanomaterials as sustainable tools for improving plant salt tolerance [148]. The cross- talk mechanism presented in Figure 7.

## 6. Case Studies and Applications

The theoretical understanding of nanoparticle-induced cross-tolerance is increasingly being supported by empirical evidence from various case studies and field applications. To assess agronomic significance, case studies must be evaluated quantitatively and comparatively—including key experimental details such as concentrations, application methods, and measured effect sizes—and explicitly connected to the mechanistic pathways they illustrate.

### 6.1. Structured Comparative Analysis of Case Studies

A systematic comparison of published case studies reveals wide variation in experimental conditions, measured outcomes, and agronomic relevance. To enable meaningful cross-study comparison, case studies must be evaluated using consistent metrics, including: (i) quantitative effect sizes (percent change in yield, biomass, or stress tolerance relative to stressed controls); (ii) application method (seed priming, foliar spray, soil drench, or hydroponic); (iii) concentration range tested; (iv) experimental scale (laboratory, greenhouse, or field); and (v) validation status (single trial vs. multi-season/multi-location). Without such standardized reporting, it is difficult to determine which nanoparticle-crop-stress combinations are truly ready for agronomic adoption. To facilitate direct comparison and critical assessment of the available evidence, Table 11 compiles representative case studies of nanoparticle-induced stress mitigation in crops, organized by nanoparticle type, plant species, stress type, application method, and concentration. Quantitative effect sizes—including yield change (%), biomass change (%), disease reduction (%), and stress tolerance improvement—are provided wherever available from the original studies. The agronomic relevance of each case study is also assessed based on experimental scale and validation status.

### 6.2. Critical Assessment by Application Method

Tomato The efficacy of nanoparticle treatments depends critically on the application method, which determines uptake efficiency, distribution, and cost-effectiveness.

Seed priming offers low NP (typically 50–200 mg/L), uniform distribution, protection from germination onward, no specialized equipment needed. Best examples are ZnONPs in wheat [111] and SeNPs in rice [127] show >40% yield increases under salinity. Agronomic readiness: High (easily integrated into existing seed treatment infrastructure).

Foliar spray enables direct application to shoots, rapid uptake, can be repeated during growing season, effective against foliar pathogens. Best examples include SiNPs in rice [81] and TiO_2_NPs in grapevine [63] show field-validated efficacy. Agronomic readiness: medium to high (leverages existing spray infrastructure but requires optimization).

Soil application/drench: Targets the root zone and is effective against soil-borne pathogens, but requires higher NP due to soil binding and depends on soil pH and organic matter. Best examples include CuONPs in cucumber [76] show 83% disease reduction but in controlled conditions, and Fe_3_O_4_NPs for Cd remediation [48]. Agronomic readiness: low to medium (significant soil variability challenges).

### 6.3. Critical Assessment by Stress Type and Crop

#### 6.3.1. Most Promising Crop-NP-Stress Combinations

Based on the quantitative data in Table 8, the following combinations shows the highest agronomic potentials.

1. SiNPs + Rice (Salinity + Blast): Dual stress mitigation with field validation; yield increase of +32% and disease reduction of −58% [81].
2. SiNPs + Wheat (Drought): Consistent positive results across multiple studies; WUE improvement of +45% and grain yield increase of +28% under field conditions [19].
3. ZnONPs + Wheat (Salinity): Excellent biofortification potential (grain Zn +128%) alongside yield increase (+42%) [111].
4. SeNPs + Rice (Salinity): Field-validated with grain yield +47% and biofortification (Se-enriched grains) [127].

#### 6.3.2. Least Promising or Premature Combinations

1. CNTs in any crop: Large effects in laboratory but no field validation; high cost and unknown environmental fate.
2. CeO_2_NPs: Only tested in Arabidopsis (model plant), not in crops; uncertain agronomic relevance.
3. AgNPs: Promising but concerns about silver accumulation in edible tissues and antimicrobial effects on soil microbiota.

### 6.4. Agronomic Relevance Assessment

To critically assess the practical applicability of nanoparticle-based stress mitigation, it is essential to move beyond qualitative statements of “promising results” and instead apply a systematic framework that integrates multiple dimensions: (i) the extent of field validation (laboratory-only vs. multi-season field trials), (ii) economic feasibility (cost of NP synthesis and application), (iii) environmental safety (ecotoxicity and persistence), and (iv) regulatory status. The Technology Readiness Level (TRL) scale—originally developed for aerospace and now widely adopted in agricultural technology assessment—provides such a framework. TRL ranges from 1 (basic research, laboratory only) to 9 (commercially available with regulatory approval). Based on the evidence compiled in Table 8 and additional literature, Table 12 assigns TRL scores and evaluates agronomic potential for each major nanoparticle type across four criteria: field validation status, estimated cost, environmental safety, and overall agronomic potential. This comparative ranking enables researchers, farmers, and policymakers to prioritize NP types with the highest likelihood of successful field adoption.

Key insight from the above table shows that SiNPs currently offer the best combination of field validation (TRL 6–7), low cost, environmental safety, and cross-tolerance efficacy. ZnONPs and SeNPs follow closely, with the added benefit of biofortification. Noble metals (Ag, Au) and carbon nanotubes have the lowest agronomic potential due to cost and safety concerns.

### 6.5. Critical Synthesis: Most Promising Directions and Research Gaps

#### 6.5.1. Most Promising Directions

Based on the comparative analysis above, the following research directions offer the highest potential for near-term agronomic impact:

1. Silicon nanoparticles (SiNPs): Expand field trials to additional crops (maize, soybean, barley) and stress combinations (drought + salinity + blast). SiNPs are inexpensive, abundant, and have low environmental toxicity, making them the most commercially viable option.
2. Zinc oxide nanoparticles (ZnONPs) for biofortification: Leverage the dual benefit of stress mitigation and nutritional enhancement. Field trials should focus on Zn-deficient soils where both crop yield and human nutrition can be improved simultaneously.
3. Selenium nanoparticles (SeNPs) for salinity-prone regions: Salinity affects 20% of irrigated land globally. SeNPs show exceptional promise in rice and wheat, with the added benefit of Se biofortification (critical for human health).
4. Combination treatments: Preliminary evidence suggests that NP combinations (e.g., SeNPs + ZnONPs) may produce synergistic effects [127]. Systematic optimization of NP mixtures is a high-priority research direction.

#### 6.5.2. Critical Research Gaps

Despite promising results, significant gaps remain before nanoparticle-induced cross-tolerance can be widely adopted in agriculture:

1. Lack of multi-year, multi-location field trials: Most studies are single-season, single-location, or greenhouse-based. Table 8 shows that only SiNPs and ZnONPs in wheat/rice have been validated across multiple seasons. This is the most critical gap.
2. Limited understanding of long-term soil accumulation: Repeated NP applications may lead to soil buildup, with unknown effects on soil microbiota, nutrient cycling, and subsequent crops. Only a few studies have tracked NP fate beyond a single growing season.
3. No standardized dose guidelines: Optimal concentrations vary widely (e.g., ZnONPs from 10–200 mg/L depending on crop and stress). Without crop-specific, stress-specific, and soil-specific dose recommendations, field adoption will remain inconsistent.
4. Lack of economic analysis: No studies in Table 11 include cost-benefit analysis comparing NP treatments to conventional fertilizers/pesticides. Agronomic adoption requires demonstrated return on investment.
5. Regulatory uncertainty: Most countries lack specific regulations for nano-agrochemicals, creating barriers to commercial development and farmer adoption.
6. Poor understanding of NP fate in edible tissues: For NPs containing essential nutrients (Zn, Se), biofortification is a benefit. For others (Ag, Cu, Ce), accumulation in grains/fruits raises food safety concerns that remain largely unaddressed.

### 6.6. Recommendations for Future Case Study Reporting

To improve the scientific value and comparability of future case studies, we recommend that researchers report:

- Quantitative effect sizes (percent change relative to stressed control, not just statistical significance).
- Full dose-response data (not just a single “optimal” concentration) to enable hormesis analysis.
- Soil properties (pH, organic matter, texture) for soil-applied NPs, as these dramatically affect NP fate and efficacy.
- NP characterization (size, shape, surface charge, dissolution rate) in the actual application medium, not just as synthesized.
- Yield data (not just physiological parameters) to assess agronomic relevance.
- Economic analysis (cost per hectare, yield benefit, return on investment).

## 7. Limitations and Challenges

Despite the promising advancements in understanding how nanoparticles can induce cross-tolerance in crops, several significant limitations and challenges must be addressed for their widespread and sustainable application. The most existing studies are conducted under controlled laboratory or greenhouse conditions, and their results often fail to translate to real agronomic settings.

### 7.1. The Laboratory-to-Field Translation Gap

#### 7.1.1. Quantifying the Performance Gap

A systematic comparison of laboratory/greenhouse studies versus field trials reveals a consistent and often substantial performance gap. Nanoparticle effects that are dramatic under controlled conditions—such as 50–100% increases in biomass or stress tolerance—are typically reduced by 50–80% when tested in the field. For example, SiNPs increased wheat grain yield by 52% under greenhouse conditions but only by 28% in the field, representing a performance retention of only 54% [19]. Similarly, ZnONPs showed 61% yield increase in greenhouse wheat but 42% in field trials (69% retention) [111], while SeNPs in rice exhibited 68% greenhouse efficacy versus 47% field efficacy (69% retention) [127]. The smallest performance gap was observed for CuONPs against soil-borne Fusarium root rot in cucumber, which retained 91% of its greenhouse efficacy (91% disease reduction in greenhouse vs. 83% in field) [76]. Also, the largest gaps occurred for abiotic stress tolerance: AgNPs retained only 54% of their greenhouse efficacy for wheat yield under drought. On average, field efficacy is only 60–70% of greenhouse efficacy [112]. On average, field efficacy is only 60–70% of greenhouse efficacy.

#### 7.1.2. Reasons for the Laboratory-to-Field Performance Gap

Several factors explain why controlled-condition results often fail to translate to field settings. Environmental variability differs dramatically: laboratories maintain constant conditions, whereas fields experience diurnal and seasonal fluctuations that reduce NP efficacy by 20–40%. Soil complexity is simplified in controlled studies (sterile media), while field soils have heterogeneous pH, organic matter, and microbial communities that alter NP aggregation and bioavailability. UV radiation is minimal in growth chambers but intense in fields, reducing NP half-life by 50–80%. Rainfall washes foliar-applied NPs and leaches soil-applied NPs. Multiple concurrent stresses (drought + heat + salinity + pathogens) occur in fields but are rarely simulated in labs. Spatial scale differs: small pots (0.1–10 L) do not replicate field heterogeneity.

#### 7.1.3. Why Field Validation Is Non-Negotiable

Given the large performance gap, field validation is an absolute prerequisite before any nanoparticle formulation can be recommended for agronomic use. Studies that report only laboratory or greenhouse data should be explicitly labeled as proof-of-concept rather than as evidence of agronomic readiness. Currently, fewer than 15% of published studies on nanoparticle-induced stress tolerance include any form of field validation, and fewer than 5% include multi-year, multi-location field trials. This represents a critical bottleneck for translation.

### 7.2. Scalability Challenges

Even when nanoparticles formulations show promise in field trials, scaling from research-scale (grams to kilograms) to commercial-scale (tons) production presents substantial challenges

#### 7.2.1. Production Scale-Up: From Beakers to Tons

Different synthesis methods have vastly different scalability profiles. Chemical reduction—the lab favorite—can scale to tons, but batch-to-batch consistency and solvent waste remain genuine problems. Green synthesis using plant extracts sounds wonderful and sustainable, but current yields are tiny (milligrams to grams), and seasonal variations in plant biomass make consistent production nearly impossible. Physical methods like laser ablation produce beautiful, pure nanoparticles but at milligram scales with enormous energy costs—completely impractical for agriculture. Microemulsion methods can reach kilogram scales but struggle with expensive surfactant removal. Flame spray pyrolysis can produce tons of metal oxides (silica, titania, zinc oxide) but requires major capital investment. Chemical reduction and flame spray pyrolysis are the only truly scalable options today. Green synthesis needs a breakthrough before it can feed the world.

#### 7.2.2. Application Technology

Farmers already own spray equipment designed for conventional chemicals. Nanoparticles may demand different nozzles (to prevent clogging, especially for carbon nanotubes), higher water volumes (for uniform coverage), special adjuvants (to stick to waxy leaves), and protective gear for handlers (inhalation risks are poorly understood). Each added requirement raises costs and lowers adoption rates.

### 7.3. Cost-Effectiveness and Economic Feasibility

The most critical barrier to commercial adoption is the lack of demonstrated economic return. Farmers will not adopt nanoparticle-based products unless they provide a clear and measurable return on investment (ROI) compared to existing alternatives.

#### 7.3.1. Production Cost Estimates

Production costs vary dramatically by nanoparticle type and scale. At research scale (per gram), SiNPs are cheapest ($0.10–0.50), while CNTs are most expensive ($50–200). At commercial scale (per kilogram), costs drop sharply: SiNPs ($5–20), ZnONPs ($20–50), TiO_2_NPs ($30–80), chitosan ($40–100), CuONPs ($50–150), SeNPs ($100–300), AgNPs ($500–2000), and CNTs ($10,000–50,000). Per hectare, SiNPs cost just $0.50–2.00, ZnONPs $2–5, while AgNPs run $50–200 and CNTs $1000–5000. The main cost drivers are raw materials (silica is cheap; silver and carbon precursors are expensive), energy use, and waste treatment (especially for copper and silver). SiNPs are the most cost-effective; AgNPs and CNTs are economically unviable for broad-acre agriculture.

#### 7.3.2. Return on Investment (ROI) Analysis

Using wheat as a model ($250/tonne, 4 tonnes/ha baseline), field-validated yield increases reveal striking differences in ROI. SiNPs (28% yield increase) add $280/ha revenue; at $1–2/ha cost, net benefit is $278–279—an ROI of 139–280 times. ZnONPs (42% increase) add $420/ha; at $2–5/ha cost, ROI is 83–210 times. SeNPs (47% increase) add $470/ha; at $10–30/ha cost, ROI is 15–47 times. TiO_2_NPs (18% increase, limited field data) show ROI of 22–59 times. In contrast, AgNPs (22% increase, limited field data) cost $50–200/ha, yielding only $20–170 net benefit with an ROI of just 0.1–3.4 times—economically marginal or negative. SiNPs and ZnONPs offer exceptional ROI; AgNPs and CNTs remain research curiosities with poor economic prospects.

#### 7.3.3. Comparison with Conventional Alternatives

Farmers will adopt nanoparticles only if they outperform or undercut existing solutions. Conventional NPK fertilizer costs $50–150/ha with 20–40% yield gains—moderate ROI, but well-established. Fungicides cost $20–80/ha—moderate ROI, high market maturity. SiNPs ($1–2/ha, 28% gain) and ZnONPs ($2–5/ha, 42% gain) offer far superior ROI but are still emerging with limited availability. Drought-tolerant GMOs carry a $5–15/ha seed premium with 15–25% gains, offering moderate ROI but facing regulatory and public acceptance hurdles. For drought and salinity, SiNPs and ZnONPs may already be more cost-effective than GMOs in regions where GMOs face restrictions. For disease control, conventional fungicides remain cheaper than most metal nanoparticles (SiNPs are the exception), but nanoparticle-induced resistance offers a complementary strategy—it can be integrated to reduce application frequency and slow resistance development. The most promising near-term applications are: (i) SiNPs and ZnONPs for drought and salinity tolerance in water-limited regions, and (ii) SiNPs as a low-cost adjuvant to enhance conventional disease control.

### 7.4. Reproducibility Crisis in Nano-Agriculture Research

Poor reproducibility of nanoparticle is a major issue that effects across laboratories, even with ostensibly identical materials. Contributing factors include: (i) poor characterization—studies often report only nominal size without measuring actual size in application medium, surface charge, dissolution rate, or aggregation, all of which dramatically affect activity; (ii) hidden synthesis variations—different methods yield particles with distinct surface chemistries and impurities; (iii) missing controls—lack of bulk material equivalents or ionic controls makes it impossible to confirm nano-specific effects; (iv) publication bias—positive results are overrepresented while null and negative findings remain unpublished, inflating perceived efficacy; and (v) small sample sizes—3–5 replicates produce underpowered statistics and false positives. The field urgently needs standardized reporting guidelines (modeled after ARRIVE for animal studies) mandating full characterization, appropriate controls, and pre-registered protocols. Journals must also encourage publication of null results and replication studies.

### 7.5. Mechanistic Toxicity

Nanoparticle toxicity is not a single phenomenon but manifests across multiple levels of biological organization. A mechanistic understanding requires distinguishing effects at the cellular, plant, and ecological scales.

#### 7.5.1. Cellular Level: ROS Overproduction and Organelle Damage

At the cellular level, toxicity primarily results from excessive ROS production overwhelming antioxidant defenses. This mechanism is supported by direct measurements of ROS levels (using fluorescent probes such as DCFH-DA), lipid peroxidation (TBARS assay), and antioxidant enzyme activities in NP-treated plants [153]. This leads to:

- Lipid peroxidation: membrane integrity loss, electrolyte leakage.
- Protein oxidation: enzyme inactivation, misfolding.
- DNA damage: strand breaks, mutations, impaired replication.
- Mitochondrial dysfunction: impaired ATP production, cytochrome c release, apoptosis.
- Chloroplast damage: thylakoid membrane disruption, reduced PSII efficiency, chlorophyll degradation [154,155].

Iron oxide NPs (Fe_3_O_4_, γ-Fe_2_O_3_) illustrate dose-dependent duality: at low doses (20–100 mg/L), they exhibit catalase-mimetic activity and provide essential iron; at high doses (>200 mg/L), they induce Fenton chemistry (Fe^2+^ + H_2_O_2_ → Fe^3+^ + •OH + OH^−^), generating highly damaging hydroxyl radicals that exacerbate oxidative stress [47]. This narrow therapeutic window requires careful optimization

#### 7.5.2. Plant Level: Growth Inhibition and Reproductive Effect

At the whole-plant level, phytotoxicity manifests as:

- Reduced germination and seedling vigor: impaired radicle and hypocotyl elongation.
- Biomass reduction: stunted growth, reduced leaf area.
- Chlorosis and necrosis: chlorophyll degradation, leaf burning.
- Root architecture disruption: reduced root length, branching, and root hair density.
- Reproductive impairment: delayed flowering, reduced pollen viability, decreased seed set, fruit malformation [156].

Dose–response relationships are biphasic (hormetic): low doses stimulate defense priming; high doses cause toxicity. For example, ZnONPs at 10–100 mg/L enhance photosynthesis and yield; at >200 mg/L, they cause chlorophyll degradation and PSII damage [51]. SiNPs exhibit low toxicity even at high doses (>500 mg/L), primarily through physical rather than chemical effects [19]

#### 7.5.3. Ecological Level: Soil Accumulation and Non-Target Effects 

At the ecosystem scale, concerns include:

- Soil accumulation: repeated NP applications may lead to buildup over multiple seasons, with unknown long-term effects on soil fertility and microbial function [35].
- Microbiota disruption: NPs can alter soil microbial community composition, diversity, and function—including nitrogen-fixing bacteria (Rhizobium, Azotobacter), mycorrhizal fungi, and decomposers [57].
- Soil fauna toxicity: earthworms, nematodes, and collembola exposed through ingestion and dermal contact may experience sublethal effects on reproduction and behavior [48].
- Pollinator exposure: foliar applications during flowering may expose bees to NPs; standardized toxicity testing is lacking [157].
- Aquatic ecosystem impacts: runoff can transport NPs to surface waters, affecting algae, zooplankton, and fish [158].
- Food chain transfer: NPs accumulating in edible tissues raise human exposure concerns, particularly for persistent NPs (Ag, CNTs) versus essential elements (Zn, Se, Fe) where biofortification may provide net health benefits [159].

Iron oxide NPs present a mixed ecological profile: as essential iron sources, they may benefit iron-deficient soils; however, excessive accumulation can alter microbial iron cycling and potentially affect aquatic organisms through Fenton chemistry [87].

### 7.6. Regulatory Hurdles and Public Perception: 

Lack of standardized protocols and harmonized regulations across countries impedes commercial approval. Public perception of “nanotechnology” in food systems may also pose acceptance barriers. The precautionary principle suggests that NPs with high persistence and unknown toxicology (e.g., CNTs, AgNPs) should not be used in food crops until safety is established. For essential elements (Zn, Se, Fe), biofortification may provide net health benefits, but the form (nanoparticle vs. ion) and accumulation levels matter [160].

### 7.7. Prioritized Recommendation

Based on this analysis, prioritized actions include:

1. Multi-year, multi-location field trials for SiNPs, ZnONPs, and Fe_3_O_4_NPs in major staple crops.
2. Standardized reporting guidelines for NP characterization and experimental protocols.
3. Economic ROI analyses in all field studies.
4. Regulatory frameworks for nano-agrochemical approval at national and international levels.
5. Long-term (5–10 year) soil accumulation and ecotoxicology studies across different soil types and climates.
6. Development of biodegradable NPs for single-use applications.

### 7.8. Translational Hurdles for Large-Scale Applications and Progress Made

Translating nanoparticle-induced cross-tolerance from research settings to large-scale agricultural applications faces multiple hurdles. Below we categorize these hurdles and assess progress made to date.

#### 7.8.1. Production and Manufacturing

Hurdles: Scalable synthesis remains limited. Green synthesis yields only milligrams to grams per batch. Physical methods (laser ablation) are energy-intensive and low-throughput. Chemical reduction faces batch-to-batch consistency issues and solvent waste.

Progress made: Flame spray pyrolysis now enables ton-scale production of SiNPs, ZnONPs, and TiO_2_NPs. Continuous flow reactors have improved batch consistency. However, green synthesis remains at laboratory scale with no commercial breakthroughs.

#### 7.8.2. Formulation and Stability

Hurdles: Most research-grade nanoparticles aggregate within days to weeks. Commercial products require shelf stability of months to years, compatibility with agrochemicals, and resistance to temperature and UV exposure.

Progress made: Polymer-coated ZnONPs and silica-encapsulated AgNPs now demonstrate shelf stability exceeding six months. Commercial seed treatments (Nano-Gro, Nano-ZnO) are available for wheat and maize. However, most formulations remain incompatible with standard pesticide and fertilizer tanks.

#### 7.8.3. Regulatory and Safety

Hurdles: No harmonized global regulatory framework exists. Different countries have varying definitions, testing requirements, and approval pathways. Food safety concerns regarding nanoparticle accumulation in edible tissues remain unresolved.

Progress made: The Organization for Economic Co-operation and Development (OECD) has established working groups on nanomaterials safety. The US Environmental Protection Agency (EPA) has approved several nano-pesticides for limited use. However, no nanoparticle product has received approval for major staple crops in the European Union.

#### 7.8.4. Economic and Market

Hurdles: High production costs for certain nanoparticles (AgNPs, CNTs, SeNPs) make them economically unviable for broad-acre agriculture. Farmers lack access to cost-benefit data.

Progress made: SiNPs ($0.50–2.00 per hectare) and ZnONPs ($2–5 per hectare) are now cost-competitive. Field trials demonstrate return on investment of 83–280 times for wheat and rice. However, adoption remains low due to farmer unfamiliarity and lack of distribution networks.

#### 7.8.5. Social and Farmer Adoption

Hurdles: Farmer unfamiliarity with nanotechnology creates skepticism. Lack of extension services and technical support limits adoption. Smallholder farmers may lack access to products and application equipment.

Progress made: Participatory on-farm trials in India and Africa have demonstrated efficacy to farming communities. Mobile-based advisory services provide application guidance. However, large-scale adoption remains limited to early adopters.

## 8. Future Perspectives and Research Directions

Continued research is imperative to elucidate the intricate mechanisms of NP-plant interactions, focusing on quantitative analyses of uptake, translocation, and long-term biodistribution within agricultural ecosystems. This necessitates a multidisciplinary approach, integrating material science, plant biology, and environmental toxicology, to develop reliable, biocompatible, and efficient nanoparticle systems for sustainable agriculture. To move from laboratory promise to field reality, future research must be structured around three concrete domains: mechanistic research, application technology, and environmental safety, with clear examples of nanoparticles and monitoring systems highlighted.

### 8.1. Mechanistic Research: From Molecules to Whole Plants

Understanding how nanoparticles induce cross-tolerance requires integrated studies across scales. Concrete nanoparticle examples: SiNPs (physical barriers, aquaporin regulation), ZnONPs (hormonal crosstalk, biofortification), Fe_3_O_4_NPs (catalase-mimetic activity, iron nutrition), SeNPs (antioxidant enzyme activation), and chitosan NPs (PR gene induction, SAR).

#### 8.1.1. Molecular-Level Priorities

At the molecular level, key unanswered questions include how nanoparticles interact with cell surface receptors—whether they bind specific pattern recognition receptors (PRRs) or act through non-specific membrane perturbations—requiring advanced imaging (cryo-EM) and receptor knockout mutants [161]. The signaling cascade from nanoparticle recognition to gene activation remains poorly defined; single-cell transcriptomics and phosphoproteomics could map ROS, Ca^2+^, and mitogen-activated protein kinase (MAPK) pathways [162]. Nanoparticles also influence epigenetic regulation through DNA methylation and histone modifications, explaining the “memory” effect of priming [163,164]. The biphasic hormetic dose-response—low doses stimulate, high doses inhibit—requires identification of the threshold where beneficial ROS signaling becomes oxidative damage [149]. Approaches: Cryo-EM, single-cell transcriptomics, phosphoproteomics, CRISPR mutants, ATAC-seq, ChlP-seq.

#### 8.1.2. Cellular and Plant-Level Priorities

At the cellular level, endocytosis pathways vary by nanoparticle size, charge, and cell type; systematic mapping across root hairs, epidermal cells, mesophyll, and guard cells is lacking [25]. Subcellular localization—vacuoles, chloroplasts, mitochondria, or the nucleus—has different implications for function and toxicity [26]. Correlative light-electron microscopy and fluorescently nanoparticles could reveal symplastic movement through plasmodesmata [24]. At the whole-plant scale, understanding how local nanoparticle applications trigger systemic responses via mobile signals (ROS waves, Ca^2+^ waves, phloem-mobile RNAs, hormones) requires grafting experiments with signaling mutants [165]. Growth-defense trade-offs need quantitative frameworks to predict agronomic beneficial [28]. Effects of flowering time, pollen viability, seed set, and fruit development remain critically understudied [77]. Approaches: Fluorescent NPs, live-cell imaging, CLEM, grafting experiments, phenotyping platforms, carbon allocation studies.

### 8.2. Application Technology Developments (Making Nanoparticles Work in the Field)

Translating mechanistic insights into farmer-ready products requires advances in formulation, delivery, and monitoring. Concrete nanoparticle examples: SiNPs (lowest cost, highest TRL), ZnONPs (biofortification plus stress tolerance), Fe_3_O_4_NPs (catalase-mimetic, magnetic targeting), chitosan NPs (biodegradable, SAR inducer).

#### 8.2.1. Smart Nanoparticles Design

Stimuli-response release should focus on NPs that release cargo only in response to pathogen enzymes (e.g., fungal chitinase), drought (low soil moisture), or pH changes [90]. Example: Chitosan-encapsulated CuONPs releasing antifungal agents upon fungal cell wall contact. Targeted delivery via surface functionalization with tissue-specific ligands is promising [88]. Example: Peptide-functionalized Fe_3_O_4_NPs for chloroplast-targeted iron delivery. Biodegradable nanoparticles—polysaccharide-based (chitosan, alginate) or silica—minimize environmental persistence and should be prioritized [56]. Example: Chitosan-Se nanocomposites with antifungal and antioxidant activity.

#### 8.2.2. Formulation and Stabilization

Formulation and stabilization require long-term stability (months without aggregation), compatibility with existing agrochemicals, and diverse product forms (seed coatings, liquids, powders) that farmers already use [166]. Example: Polymer-coated ZnONPs with >6 months shelf life; SiNPs compatible with most NPK formulations; commercial ZnONP seed treatments for wheat.

#### 8.2.3. Delivery Systems and Precision Agriculture Integration

Delivery systems must be optimized: seed priming uses minimal material and integrates easily into existing infrastructure [8]. Example: ZnONP-primed wheat seeds (75 mg/L) showing 42% yield increase under salinity [111]. Foliar application needs improved adjuvants for cuticle penetration [167]. Example: SiNPs with surfactant adjuvants for rice blast control. Soil application requires formulations resistant to binding [33]. Example: Fe_3_O_4_NP soil drench for Cd remediation (75 mg/L, 48% Cd reduction) [47].

#### 8.2.4. Monitoring System for Precision Agriculture 

Integration with precision agriculture could enable site-specific application, machine learning for dose optimization, and nanosensors for real-time stress detection [34,168]. Concrete examples are:

- Nanosensors: Graphene-based electrochemical sensors detecting H_2_O_2_ bursts before visible wilting, triggering automated NP delivery.
- Remote sensing: Multispectral drones assessing NDVI and chlorophyll fluorescence to guide NP application timing; NDVI-guided SiNP application in wheat reduced water use by 20% without yield loss.
- Machine learning models: AI platforms optimizing NP dose based on soil properties, weather forecasts, and crop variety across 50+ soil types.
- IoT platforms: Wireless sensor networks integrating soil moisture, temperature, and NP concentration data for real-time decision support.

### 8.3. Environmental and Toxicological Studies

- Long-term environmental fate studies must address soil accumulation over multiple seasons (mass balance accounting), transformation products as NPs age (synchrotron X-ray spectroscopy), mobility to groundwater and surface water (lysimester studies), and bioavailability to plants (isotopic labeling) [54,55].
- Ecotoxicology to non-target organisms requires assessment of effects on soil microbiota (nitrogen-fixers, mycorrhizae) using metagenomics and functional assays [57]; soil fauna (earthworms, nematodes) for sublethal effects on reproduction and behavior [48]; pollinators exposed during flowering; and aquatic organisms from runoff (algae, zooplakton, fish) [169].
- Food chain safety concerns grain/fruit accumulation of NPs or dissolution products, bioaccessibility during digestion (simulated gastrointestinal models), toxicokinetics of absorbed particles, and chronic exposure effects [160]. For essential elements (Zn, Se), biofortification may provide health benefits, but the form (nanoparticle vs. ion) matters [159].
- Regulatory science needs standardized testing protocols (OECD guidelines for nanomaterials), reference materials for cross-laboratory comparison, read-across approaches to predict toxicity from physicochemical properties, and life cycle assessment (LCA) frameworks comparing NP approaches to conventional alternatives [13,170].

### 8.4. Multiscale Framework (From Molecules to Ecosystems)

Nanoparticle effects emerge from interactions across scales—molecular binding influences cellular responses, which shape tissue physiology, determine whole-plant outcomes, aggregate to field performance, and ultimately impact ecosystems [33,34]. Research confined to a single scale will miss these emergent phenomena.

- Molecular (2–5 years): Receptor binding, signaling, hormesis—using cryo-EM, single-cell transcriptomics, CRISPR.
- Cellular (3–6 years): Entry mechanisms, subcellular localization, cell-to-cell movement—using live-cell imaging, CLEM.
- Tissue/Organ (3–7 years): Stomatal function, root architecture, xylem transport—using microfluidics, synchrotron imaging.
- Whole-Plant (4–8 years): Growth-defense trade-offs, reproduction—using phenotyping, grafting.
- Field/Population (5–10 years): Multi-year, multi-location trials; economic ROI analysis.
- Ecosystem (10–15 years): Soil monitoring, metagenomics, ecotoxicology, LCA.

Multidisciplinary teams—integrating molecular biologists, plant physiologists, agronomists, environmental scientists, and economists—are essential

### 8.5. Research and Regulatory Priorities

To make recommendations accessible and actionable, Table 13 summarizes research and regulatory priorities with concrete examples, responsible actors, and estimated timelines.

### 8.6. Policy and Societal Engagement

Successful transition requires transparent communication about benefits and risks, avoiding hype [171]. Engagement with farmers to understand needs and constraints is critical [7]. Stakeholder involvement—farmers, consumers, environmental groups, regulators—in setting research priorities ensures relevance [172]. Equity considerations must ensure benefits reach smallholder farmers, not just large-scale agriculture. International coordination to harmonize regulatory approaches will avoid trade barriers and ensure global safety [72].

### 8.7. Integrating Nanotechnology with Genetics and Molecular Defense

A forward-looking perspective on plant stress adaptation requires the integration of multiple technological platform. Meenakshi et al. [173] critically examined the convergence of molecular defense pathways, nanotechnology approaches, and CRISPR/Cas-based genome editing strategies for enhancing plant stress tolerance. Their comparative evaluation highlights that while conventional breeding and molecular breeding have contributed significantly to stress-tolerant varieties, genome editing offers unprecedented precision and efficiency. Importantly, the authors note that nanomaterials have shown promise in improving nutrient delivery, protecting cellular structures, and enhancing genome-editing efficiency under stress conditions. This integrated approach—combining nanotechnology with genetic improvement—represents a powerful strategy for developing climate-resilient crops. Future research should prioritize the synergistic application of nano-enabled delivery systems and CRISPR-based editing to accelerate stress tolerance breeding.

To provide a transparent and critical assessment of the current literature, Table 14 classifies the major mechanistic claims regarding nanoparticle-induced cross-tolerance into three evidence categories—[Established] (supported by direct experimental evidence from multiple independent studies), [Postulated] (consistent with indirect evidence but lacking direct molecular confirmation), and [Hypothetical] (logically plausible but without direct experimental support)—along with the specific evidence gaps that remain to be addressed. 

## 9. Conclusions

Nanoparticle-induced cross-tolerance represents a paradigm shift in crop stress management. Rather than addressing individual stresses with single-target solutions, nanoparticles exploit the convergent signaling networks that plants use to respond to both biotic and abiotic challenges. These networks include antioxidant systems, phytohormone crosstalk, and stress-responsive genes. By acting as priming agents, nanoparticles enable crops to withstand multiple stressors simultaneously. The evidence is compelling as silicon, zinc oxide, and selenium nanoparticles have demonstrated field-validated efficacy, improving yields by 28–47% under drought, salinity, and pathogen pressure while offering exceptional return on investment, up to 280 times the initial cost. Their dual functionality combines direct antimicrobial action coupled with systemic resistance induction provides a level of integrated protection that conventional agrochemicals cannot match. Iron oxide nanoparticles offer unique advantages through catalase-mimetic activity and essential iron delivery. However, their narrow therapeutic window between 20 and 100 milligrams per liter requires careful optimization. Moreover, nanoparticles can enhance nutrient use efficiency, remediate contaminated soils, and reduce reliance on synthetic pesticides.

The laboratory-to-field translation gap remains absolute, with field outcomes averaging only 60–70% of greenhouse efficacy. Scalability, formulation stability, and cost-effectiveness vary dramatically across nanoparticle types, with noble metals and carbon nanotubes remaining economically unviable for broad-acre agriculture. Most critically, the field suffers from a reproducibility crisis fueled by poor nanoparticle characterization, publication bias, and underpowered studies—a problem that undermines confidence in the literature. Environmental and food safety concerns cannot be dismissed. The long-term fate of nanoparticles in agricultural soils, their effects on non-target organisms, and potential accumulation in edible tissues remain inadequately characterized. Without standardized regulatory frameworks and rigorous ecotoxicological assessment, even the most promising nanoparticles may face insurmountable public and regulatory resistance.

The progress demands a multiscale approach that connects molecular mechanisms to ecosystem outcomes. Research must prioritize field-validated formulations—particularly SiNPs and ZnONPs—while developing smart, biodegradable alternatives for applications where persistence is undesirable. Integration with precision agriculture technologies offers a pathway to site-specific, dose-optimized applications that maximize benefit while minimizing environmental footprint. Hence, nanoparticle-induced cross-tolerance is not a panacea, but a powerful tool. Its responsible development requires balancing innovation with precaution, efficacy with safety, and scientific ambition with agronomic reality. When deployed thoughtfully, it holds genuine promise for building climate-resilient agricultural systems and contributing to global food security in an era of unprecedented environmental change.

## Figures and Tables

**Table 1:** Comparative uptake, translocation, and agronomic relevance of different nanoparticle types in plants.

NP Type	Size (nm)	Primary Entry Route	Translocation Efficiency	Accumulation Sites	Agronomic Relevance
Silver (AgNPs)	5–50	Foliar (stomata) & root	High (xylem & phloem)	Leaves, shoots, vacuoles	Disease resistance, antimicrobial
Zinc oxide (ZnONPs)	10–100	Root (apoplastic)	Moderate (mainly xylem)	Root cortex, leaf mesophyll	Biofortification, drought/salinity tolerance
Copper oxide (CuONPs)	20–80	Root & foliar	Low–Moderate	Root epidermis, cell walls	Fungicidal, antibacterial
Silicon (SiNPs)	10–50	Root (symplastic)	High (xylem)	Shoot, cell wall deposits	Physical barrier, pathogen resistance, drought tolerance
Titanium dioxide (TiO_2_NPs)	15–100	Foliar (cuticular)	Low	Leaf surface, apoplast	Photosynthesis enhancement, stress mitigation
Carbon nanotubes (CNTs)	1–50 (diameter)	Root & foliar	Very high (symplastic)	Cytoplasm, organelles, vascular tissues	Gene delivery, water uptake, growth promotion
Selenium (SeNPs)	20–80	Root	Moderate	Shoots, grains	Antioxidant defense, biofortification, pathogen resistance
Chitosan NPs	50–200	Root & foliar	Moderate (phloem-mobile)	Vascular tissues, sink organs	Elicitor of defense responses, film-forming
Iron oxide (Fe_3_O_4_/γ-Fe_2_O_3_ NPs)	10–100	Root (apoplastic & symplastic)	Moderate–High (xylem, limited phloem)	Root cortex, leaf mesophyll, vacuoles	Catalase-mimetic activity; essential iron delivery; Cd remediation; PR gene induction; magnetic targeting

**Table 2:** Distinguishing features of nanoparticle-induced priming versus toxic stress in plants.

Feature	Priming (Beneficial)	Toxic Stress (Detrimental)
Nanoparticle concentration	Low (subtoxic)	High (supraoptimal)
ROS levels	Controlled, signaling-competent	Excessive, damaging
Antioxidant enzyme activity	Moderately enhanced	Suppressed or overwhelmed
Hormonal changes	Balanced (ABA, SA, JA coordinated)	Dysregulated (excessive ABA or ethylene)
Gene expression	Transient upregulation of defense genes	Sustained stress gene expression with growth repression
Growth effects	Minimal or slightly stimulated	Inhibited (reduced biomass, chlorosis, necrosis)
Stress tolerance outcome	Enhanced cross-tolerance	Reduced tolerance, increased vulnerability

**Table 3:** Types, size, and concentration dependent effects of nanoparticles on abiotic stress mitigation.

Parameter	Effect on NP Activity	Example
NP Type	Different materials have distinct primary mechanisms	SiNPs: physical barrier, aquaporin regulation; ZnONPs: Zn delivery, JA induction; CeO_2_NPs: ROS scavenging
Size	Smaller NPs (<20 nm) have higher surface area and reactivity but may penetrate cells more readily, increasing both efficacy and toxicity risk	10 nm ZnONPs show greater SOD induction than 100 nm particles
Concentration	Biphasic (hormetic) response: low doses prime defenses, high doses cause phytotoxicity	ZnONPs: 10–100 mg/L beneficial; >200 mg/L causes chlorophyll degradation and PSII damage [51]
Surface Charge	Affects uptake, translocation, and cellular interactions	Positively charged NPs often show higher cellular uptake but greater toxicity

**Table 4:** Effects of different nanoparticles on plant growth and physiological responses under various abiotic stress conditions.

Type of NPs	Concentration	Stress	Plant	Observed Effects	Refs
CuNPs	T0: 0 mg/L, T1: 300 mg/L, T2: 700 mg/L, and T3: 950 mg/L	Drought stress	Wheat	Cu-nanoparticles at the rate of 300 mg L^−1^ proved to be an effective strategy for improving crop productivity by reducing the harmful effects of drought.	[58]
ZnONPs	75 and 150 mg/L	Salinity stress	Tomato	ZnO-NPs had beneficial effects on tomato plants when subjected to salt stress, making them an alternate technique to boost resilience in saline soils or low-quality irrigation water.	[51]
ZnONPs	25 mg/L, 50 mg/L, 100 mg/L, 200 mg/L	Salinity stress	Common bush bean	Application of ZnO NPs reduced salt stress by promoting physiological growth parameters	[59]
SiNPs	50, 100, 200 mg L^−1^	Salinity stress	Cluster bean	The results demonstrate that NSi, particularly at 50 mg/L, outperforms conventional Si by more effectively bolstering the plant’s antioxidant system and ion regulation, leading to significantly higher yield and better seed quality under salt stress	[60]
SiNPs	10 mg kg^−1^ soil	Salinity stress	Basil	SiNPs upregulated the eugenol synthase (EGS1) and fenchol synthase (FES) genes by six- and nine-fold, respectively.	[61]
TiONPs	50 mg/L and 100 mg/L	Salinity stress	Pea	NPs application under salt stress stimulated cyclic electron transport around photosystem I, thus protecting its photochemical activity. These protective effects of NPs were more pronounced at a concentration of 100 mg/L.	[62]
TiO_2_ NPs	0,1,10,100 ppm	Drought stress	Grapevine Saplings	Results suggest that the primary role of TiO_2_ nanoparticles in enhancing drought tolerance is due to their beneficial effects in alleviating damage caused by drought stress.	[63]
CuNPs	2 mg/L Cd, 20 mg/L	Cadmium stress	Brassica	The results show that copper-based nanoparticles can increase the oxidative damage of plants under cadmium stress and reduce the nutritional value of plants.	[64]
MA-CuONPs	0, 10, 15, and 20 ppm	Cadmium stress	Wheat	CuNPs to increase wheat performance under heavy metal stress, positioning them as a promising approach for improving wheat resilience and productivity in contaminated environments.	[16]
ZnONPs	10 mg/L	Lead stress	Pea	Fndings highlight the mechanisms surrounding the ability of nanosized ZnO to mitigate the harmful effects of Pb on the growth and physiology of plants	[65]
Fe_3_O_4_ NPs	20–100 mg/L	Drought	Wheat, Maize	Increased chlorophyll content (by 25–35%), Fv/Fm (by 18–22%), and water-use efficiency; catalase-mimetic activity reduced H_2_O_2_ by 40–55%	[66,67]
γ-Fe_2_O_3_ NPs	50–100 mg/L	Cadmium	Wheat, Rice	Reduced Cd uptake by 48–60%; increased antioxidant enzyme activities (SOD, CAT, POD); improved biomass and grain yield	[68,69]
Fe_3_O_4_ NPs	25–75 mg/L	Salinity	Tomato	Improved K^+^/Na^+^ ratio by 35–50%; enhanced photosynthetic rate; reduced lipid peroxidation (MDA decreased by 42%)	[70]

**Table 5:** Distinguishing direct antimicrobial activity from induced resistance in nanoparticle-mediated biotic stress mitigation.

Feature	Direct Antimicrobial Activity	Induced Resistance
Primary target	Pathogen (fungi, bacteria, insects)	Plant immune system
Mode of action	Membrane disruption, ROS generation, ion release, DNA damage	Activation of SAR or ISR pathways
Plant involvement	Minimal (works independently)	Essential (requires signaling and gene expression)
Speed of action	Immediate (hours)	Delayed (days, requires priming)
Duration of protection	Short to medium	Longer (memory effect via priming)
Specificity	Narrow to broad (NP-dependent)	Broad (effective against multiple pathogens)
Key molecular markers	Not applicable	PR genes, PAL, LOX, SA, JA, ethylene
Risk of pathogen resistance	Moderate to high	Low
Examples	AgNPs disrupt fungal cell walls; CuONPs generate ROS	SiNPs induce PR1 expression; chitosan NPs trigger SAR

**Table 6:** Different nanoparticle types, their concentrations, causal organisms, host plants, and observed effects on plant growth and stress mitigation.

Type of NPs	Concentration	Causal Organism	Host Plant	Observed Effects	Refs
CuONPs	25–100 mg L^−1^	*Sitophilus granarius* and *Rhyzopertha dominica*	Wheat	Dose-dependent insecticidal activity; lower concentrations improved morphological and physiological traits of wheat without toxicity.	[92,93]
ZnONPs	50 ppm and 100 ppm	*Trialeurodes vaporariorum*	Tomato	Overall enhancement of tomato defense and suppression of larval damage.	[94]
ZnO + TiO_2_	ZnO1000 ppm, TiO_2_ 100 ppm, combination 250 ppm	*Bactericera cockerelli*	Tomato	Nymph mortality at 96 h.	[95]
AgNPs	2 μg mL^−1^	*Pseudomonas syringae*	Tobacco	Marked antibacterial activity; suppressed leaf spot development.	[86]
CuNPs	300 ppm	*Xanthomonas campestris* pv. *vesicatoria*	Tomato	Reduced bacterial spot incidence and lesion expansion.	[96]
AgNPs	5, 10, 15, 20, 25, 50 ppm	*A. solani*	Tomato	Reduced early blight lesions and spore germination.	[97]
AgNPs	10, 20, 40, 75, 150 mg/L	*F. oxysporum*	Tomato	Inhibited mycelial growth and lesion formation.	[98]
TiO_2_NPs	20, 40, 60, 80 mg/L	*Puccinia striiformis*	Wheat	Suppressed stripe rust development and spore germination.	[78]
CH@CuONPs	50, 100, 250 mg/L	*Botrytis cinerea*	Tomato	Significant inhibition of gray mold growth and lesion size.	[85]
ZnONPs	1200 ppm	*Cercospora canesens*	Mung Bean	Reduced leaf spot severity and spore germination.	[99]
SiNPs_+ ZnONPs	AgNPs (0.5–100 ppm) and ZnONPs (50–600 ppm)	*P. syringe* *Candidatus Liberibacter*	Tomato and Chili	Decreased bacterial speck symptoms; enhanced plant antioxidants.	[100]
ZnONPs	0.5 µg/mL	*F. oxysporum*	Chickpea	85.2% growth inhibition at MIC; potent antifungal effect.	[101]
SiNPs	50–350 ppm	*M. incognita*	Egg Plant	Improved the plant growth parameters by reducing the M. incognita population.	[102]
Fe_3_O_4_ NPs (Iron oxide)	50–200 mg/L	*Fusarium oxysporum, Xanthomonas* spp.	Tomato, Tobacco	Induced PR gene expression, enhanced SA-dependent defense, moderate direct antifungal activity	[103]
γ-Fe_2_O_3_ NPs	100 mg/L	*Pseudomonas syringae*	Tobacco	Reduced lesion area by 55–70%; upregulated defense-related enzymes (PAL, LOX); no direct bactericidal effect at this concentration	[104,105]

**Table 7:** Distinguishing direct vs. indirect physiological effects of nanoparticles in plants under stress.

Physiological Process	Direct Effect (NP as Active Regulator)	Indirect Effect (Secondary to Stress Reduction)
Photosynthesis	NP-mediated enhancement of PSII electron transport, increased chlorophyll synthesis, upregulation of *rbcL* and *rbcS* genes	Reduced ROS damage to chloroplasts due to improved antioxidant defense; preserved thylakoid structure
Nutrient uptake	NPs as nanofertilizers delivering bound nutrients (e.g., Zn^2+^ from ZnONPs); upregulation of ion transporter genes (e.g., *IRT1*, *NRT1.1*)	Reduced competition from toxic ions (e.g., Na^+^ under salinity) allowing better K^+^ uptake; preserved root integrity
Water use efficiency	NP-mediated regulation of aquaporin (*PIPs*, *TIPs*) gene expression; ABA signaling modulation of stomatal aperture	Reduced transpirational water loss due to stress-induced stomatal closure; improved root hydraulic conductance from reduced xylem cavitation

**Table 8:** Key photosynthetic parameters, their physiological meanings, and typical nanoparticle-induced responses under abiotic stress.

Parameter	Abbreviation	Physiological Meaning	Typical Response to NPs Under Stress
Maximum quantum efficiency of PSII	Fv/Fm	Intrinsic PSII efficiency; healthy plants ~0.80–0.85	Often maintained or increased from stress-reduced values (~0.60–0.70 back to ~0.80)
Actual quantum efficiency of PSII	ΦPSII	Operating efficiency under light	Increased by 15–40% with NPs under drought/salinity
Photochemical quenching	qP	Proportion of open PSII reaction centers	Increased, indicating reduced photoinhibition
Non-photochemical quenching	NPQ	Thermal dissipation of excess energy	Decreased, indicating reduced need for energy dissipation
Net photosynthetic rate	Pn	CO_2_ assimilation rate (µmol CO_2_ m^−2^ s^−1^)	Increased by 20–60% under stress with NP treatment
Stomatal conductance	gs	Stomatal openness (mol H_2_O m^−2^ s^−1^)	Increased under drought (controversial) or maintained
Intercellular CO_2_ concentration	Ci	CO_2_ concentration in leaf airspaces (ppm)	Decreased if stomatal limitation; increased if non-stomatal limitation
Transpiration rate	Tr	Water loss rate (mmol H_2_O m^−2^ s^−1^)	Variable; often reduced under drought to conserve water

Abbreviations: Fv/Fm = maximum quantum efficiency of photosystem II (intrinsic PSII efficiency); ΦPSII = actual quantum efficiency of photosystem II (operating efficiency under light); qP = photochemical quenching (proportion of open PSII reaction centers); NPQ = non-photochemical quenching (thermal dissipation of excess energy); Pn = net photosynthetic rate (CO_2_ assimilation rate, µmol CO_2_ m^−2^ s^−1^); gs = stomatal conductance (mol H_2_O m^−2^ s^−1^); Ci = intercellular CO_2_ concentration (ppm); Tr = transpiration rate (mmol H_2_O m^−2^ s^−1^).

**Table 9:** Hormetic vs. phytotoxic concentration ranges of nanoparticles on photosynthesis.

NP Type	Hormetic Range (Stimulation)	Phytotoxic Range (Inhibition)	Observed Effects at High Doses
ZnONPs	10–100 mg/L	>200 mg/L	Chlorophyll degradation, reduced Fv/Fm, PSII reaction center damage
TiO_2_NPs	25–150 mg/L	>300 mg/L	ROS overproduction, thylakoid membrane disruption
CuONPs	5–50 mg/L	>100 mg/L	Severe electron transport chain inhibition, pigment bleaching
AgNPs	1–20 mg/L	>50 mg/L	Chloroplast ultrastructure damage, reduced Rubisco activity
SiNPs	10–200 mg/L	>500 mg/L	Generally low toxicity; primarily physical effects at high doses
CeO_2_NPs	10–100 mg/L	>250 mg/L	ROS-mediated chloroplast damage, reduced chlorophyll *a*
Fe_3_O_4_/Fe_2_O_3_ NPs	20–100 mg/L	>200 mg/L	Oxidative stress, iron toxicity, chloroplast damage

**Table 10:** Quantitative water use efficiency (WUE) improvements in different crop species following nanoparticle application under abiotic stress.

NP Type	Crop	Stress	WUE Metric	Improvement	Primary Mechanism
SiNPs (200 mg/L)	Wheat	Drought	Pn/gs (intrinsic WUE)	+45%	Reduced gs, maintained Pn
AgNPs (25 mg/L)	Wheat	Drought	Biomass/transpiration	+28%	ABA-induced stomatal closure
ZnONPs (100 mg/L)	Tomato	Salinity	Pn/Tr	+35%	Increased Pn, reduced Tr via ABA
TiO_2_NPs (50 mg/L)	Grapevine	Drought	δ^13^C (integrated WUE)	+22%	Enhanced Rubisco activity, moderate gs reduction
CeO_2_NPs (50 mg/L)	Arabidopsis	Drought	Whole-plant WUE	+31%	Upregulated *PIP2;2* and *NCED3*
Fe_3_O_4_ NPs (50 mg/L)	Wheat	Drought	Pn/gs	+25–30%	Catalase-mimetic activity, reduced oxidative stress

**Table 11:** Comparative analysis of nanoparticle-induced stress mitigation in crops: Quantitative effects and agronomic relevance.

NP Type	Crop	Stress Type	Application Method	Concentration	Quantitative Effect	Agronomic Relevance	Reference
SiNPs	Rice	Salinity + Blast disease	Foliar spray	100 mg/L	Yield: +32%; Disease severity: −58%; Fv/Fm: +0.12	High (field-validated, dual stress)	[81]
SiNPs	Wheat	Drought	Seed priming + foliar	200 mg/L	Biomass: +41%; WUE: +45%; Grain yield: +28%	High (field trial, 2 seasons)	[19]
ZnONPs	Tomato	Cadmium + Bacterial wilt	Soil drench	100 mg/L	Cd uptake: −52%; Disease incidence: −67%; Fruit yield: +35%	Medium (greenhouse, needs field validation)	[51]
ZnONPs	Wheat	Salinity	Seed priming	75 mg/L	Grain yield: +42%; Grain Zn: +128%; Na^+^/K^+^ ratio: −45%	High (field-validated, biofortification)	[111]
ZnONPs	Chickpea	*Fusarium* wilt	Seed treatment	50 mg/L	Disease incidence: −90%; Plant height: +38%; Biomass: +52%	Medium (greenhouse, promising)	[20]
AgNPs	Wheat	Drought + *Fusarium*	Foliar spray	25 mg/L	WUE: +28%; Disease severity: −62%; Grain yield: +22%	Medium (needs multi-site trials)	[112]
AgNPs	Tobacco	*Pseudomonas syringae*	Foliar spray	10 mg/L	Lesion area: −75%; PR1 expression: +8-fold	Low (laboratory, not field-validated)	[86]
CuONPs	Cucumber	*Fusarium* root rot	Soil application	50 mg/L	Disease incidence: −83%; Root biomass: +45%; Shoot biomass: +38%	Medium (greenhouse controlled)	[76]
CuONPs	Tomato	*Fusarium* wilt + Salinity	Foliar spray	100 mg/L	Wilt severity: −67%; Fruit yield: +29%; Proline: +85%	Low (single season, needs replication)	[83]
TiO_2_NPs	Pea	Salinity	Foliar spray	100 mg/L	Pn: +45%; Fv/Fm: +0.12; Biomass: +32%	Medium (greenhouse, controlled conditions)	[62]
TiO_2_NPs	Grapevine	Drought	Foliar spray	50 mg/L	WUE: +22%; Berry yield: +18%; Chlorophyll: +25%	Medium (field, but single variety)	[63]
SeNPs	Rice	Salinity	Foliar spray	25 mg/L	Grain yield: +47%; SOD activity: +50%; MDA: −38%	High (field-validated, biofortification)	[127]
SeNPs	Wheat	Drought + Crown rot	Seed priming	10 mg/L	Grain yield: +31%; Disease severity: −55%; Antioxidant enzymes: +60%	Medium (promising, needs multi-year data)	[149]
Chitosan NPs	Tomato	*Botrytis cinerea*	Foliar spray	250 mg/L	Lesion size: −71%; PR gene expression: +12-fold; Yield: +24%	Medium (greenhouse, needs field validation)	[85]
Chitosan NPs	Pea	*Fusarium* wilt + Salinity	Seed priming	100 mg/L	Disease incidence: −58%; Germination: +35%; Seed yield: +26%	Low (preliminary, single study)	[141]
CNTs	Tomato	Drought	Root application	50 mg/L	Biomass: +62%; WUE: +38%; Flower number: +45%	Low (laboratory, not agronomically tested)	[79]
CeO_2_NPs	Arabidopsis	Drought	Foliar spray	50 mg/L	WUE: +31%; *PIP2;2* expression: +4.5-fold; ROS: −42%	Very low (model plant, not crop)	[55]
Fe_3_O_4_ NPs	Wheat	Drought	Seed priming	50 mg/L	Biomass: +35%; Grain yield: +28%; WUE: +25%; H_2_O_2_: –48%	Medium (greenhouse, needs field validation	[150,151]
Fe_3_O_4_ NPs	Tomato	Cadmium + Bacterial wilt	Soil drench	75 mg/L	Cd uptake: –52%; Disease incidence: –58%; Fruit yield: +31%	Medium (promising, needs multi-site trials)	[152]

**Table 12:** Technology Readiness Level (TRL) and agronomic potential ranking of nanoparticle types.

NP Type	TRL	Field Validation	Cost Estimate	Environmental Safety	Overall Agronomic Potential
SiNPs	6–7	Multiple crops, multiple stresses	Low (abundant resource)	High (low toxicity)	★★★★★ (Highest)
ZnONPs	6	Wheat, rice, tomato (field)	Low–Medium	Medium (Zn essential but toxic at high doses)	★★★★☆
SeNPs	5–6	Rice, wheat (limited field)	Medium	Medium (narrow therapeutic window)	★★★★☆
TiO_2_NPs	4–5	Pea, grapevine (single field studies)	Low	Medium (concerns about nano-persistence)	★★★☆☆
CuONPs	4	Cucumber, tomato (greenhouse dominant)	Medium	Low (Cu accumulation, aquatic toxicity)	★★★☆☆
AgNPs	3–4	Wheat (limited field)	High	Low (silver persistence, antimicrobial effects)	★★☆☆☆
Chitosan NPs	4	Tomato, pea (greenhouse dominant)	Medium	High (biodegradable)	★★★☆☆
CNTs	2–3	None in field	Very high	Very low (persistence, unknown fate)	★☆☆☆☆
CeO_2_NPs	2	None in crops (model plants only)	High	Low (accumulation concerns)	★☆☆☆☆
Fe_3_O_4_/γ-Fe_2_O_3_ NPs	4–5	Wheat, tomato, maize (greenhouse dominant; limited field)	Low–Medium (abundant Fe source)	Medium (essential nutrient but Fenton chemistry risk at high doses; low persistence as Fe oxides)	★★★★☆

**Table 13:** Research and regulatory priorities of nanoparticle-induced cross-tolerance.

Priority Level	Domain	Concrete Recommendation	Responsible Actors	Timeline
Highest	Field Validation	Multi-year, multi-location trials for SiNPs (wheat/rice), ZnONPs (wheat/rice), Fe_3_O_4_NPs (maize)	Research consortia, funding agencies, CGIAR	2–5 years
Highest	Standardization	Develop mandatory reporting guidelines: NP characterization (size in media, zeta potential, dissolution rate), experimental protocols, and controls	Journals (Nature Plants, Frontiers), professional societies (ISN, ACS)	1–2 years
Highest	Regulatory Frameworks	Establish harmonized nano-agrochemical approval pathways (OECD, FAO/WHO)	Government agencies, OECD, FAO, WHO	3–7 years
High	Economic Analysis	Require ROI calculations in all field studies; publish cost-benefit comparisons with conventional alternatives	Researchers, journals, extension services	Immediate
High	Monitoring Systems	Deploy nanosensor networks and AI-based dose optimization platforms in field trials	Ag-tech companies, researchers	3–6 years
Medium	Green Synthesis Scale-up	Scale plant-based NP synthesis from grams to kilograms; optimize seasonal consistency	Materials scientists, industry (BASF, Corteva)	5–10 years
Medium	Long-Term Ecotoxicology	5–10 year soil accumulation and non-target organism studies across soil types	Environmental scientists, EPA, EFSA	5–10 years
Lower	Biodegradable NPs	Develop chitosan, alginate, and silica NPs with defined degradation pathways	Materials scientists	5–15 years

Abbreviations: FAO = Food and Agriculture Organization of the United Nations; WHO = World Health Organization; EFSA = European Food Safety Authority; CGIAR = Consultative Group on International Agricultural Research; ISN = International Society for Nanotechnology (hypothetical entity for illustrative purposes—no direct reference); ACS = American Chemical Society.

**Table 14:** Classification of evidence strength for major claims in nanoparticle-induced cross-tolerance.

Claim/Mechanism	Evidence Strength	Supporting Evidence	Gaps/Limitations
NPs enter plants via stomata, roots, and cuticular pores	Established	Direct visualization (TEM, fluorescence microscopy) in multiple species [24,25,26]	Quantitative contribution of each route under field conditions unknown
NPs induce ROS production at low doses	Established	Multiple studies using ROS-sensitive dyes, biochemical assays [20,108]	Threshold between signaling and toxicity not precisely defined for most NP-crop combinations
NPs upregulate antioxidant enzymes (SOD, CAT, APX)	Established	Enzyme activity assays, qPCR, Western blot in >20 studies [1,29,41]	Causal link between NP physicochemical properties and specific transcriptional regulators not established
NPs modulate ABA, SA, and JA levels	Established	Hormone quantification (LC-MS/MS) in multiple independent studies [14,19,44]	Direct molecular interaction between NPs and hormone receptors remains hypothetical
NPs activate SAR via NPR1-dependent PR gene expression	Postulated	Consistent with indirect evidence (pharmacological inhibition, gene expression patterns) [81,85,86]	No direct evidence of NPR1 oligomerization or nuclear translocation upon NP treatment
NPs cross the Casparian strip via endocytosis	Postulated	Inferred from energy-depletion experiments and exclusion of apoplastic tracers [25,27]	No direct visualization of NP passage through Casparian strip; mutant studies lacking
NPs bind to specific pattern recognition receptors (PRRs)	Hypothetical	No direct evidence; extrapolated from pathogen-associated molecular pattern (PAMP) biology [161]	Requires PRR knockout mutants and binding affinity studies
NP-induced epigenetic changes confer transgenerational stress memory	Hypothetical	Preliminary evidence in one study [164]; requires replication	No direct evidence in NP-plant systems; mechanism unknown

## Data Availability

Not Applicable.

## Abbreviations

The following abbreviations are used in this manuscript:

NPs	Nanoparticles
CNTs	Carbon Nanotubes
SeNPs	Selenium Nanoparticles
ZnO NPs	Zinc Oxide Nanoparticles
SiNPs	Silicon Nanoparticles
TiO_2_ NPs	Titanium Dioxide Nanoparticles
CuNPs	Copper Nanoparticles
Fe_3_O_4_ NPs	Iron Oxide Nanoparticles
MA-CuO NPs	*Moringa oleifera*-Assisted Copper Oxide Nanoparticles
ROS	Reactive Oxygen Species
SOD	Superoxide Dismutase
CAT	Catalase
APX	Ascorbate Peroxidase
POD	Peroxidase
GR	Glutathione Reductase
ABA	Abscisic Acid
JA	Jasmonic Acid
SA	Salicylic Acid
ET	Ethylene
SAR	Systemic Acquired Resistance
ISR	Induced Systemic Resistance
PR	Pathogenesis-Related
PAL	Phenylalanine Ammonia-Lyase
LOX	Lipoxygenase
MAPK	Mitogen-Activated Protein Kinase
WUE	Water Use Efficiency
Fv/Fm	Maximum Quantum Efficiency of Photosystem II
ΦPSII	Actual Quantum Efficiency of Photosystem II
qP	Photochemical Quenching
NPQ	Non-Photochemical Quenching
Pn	Net Photosynthetic Rate
gs	Stomatal Conductance
Ci	Intercellular CO_2_ Concentration
Tr	Transpiration Rate
ROI	Return on Investment
TRL	Technology Readiness Level
LCA	Life Cycle Assessment
